# Deconvolution of calcium imaging data using marked point processes

**DOI:** 10.1371/journal.pcbi.1007650

**Published:** 2020-03-12

**Authors:** Ryohei Shibue, Fumiyasu Komaki

**Affiliations:** 1 NTT Communication Science Laboratories, Atsugi-shi, Japan; 2 Department of Mathematical Informatics, The University of Tokyo, Tokyo, Japan; 3 RIKEN Center for Brain Science, Wako-shi, Japan; 4 International Research Center for Neurointelligence (IRCN), The University of Tokyo, Tokyo, Japan; University of Toronto at Scarborough, CANADA

## Abstract

Calcium imaging has been widely used for measuring spiking activities of neurons. When using calcium imaging, we need to extract summarized information from the raw movie beforehand. Recent studies have used matrix deconvolution for this preprocessing. However, such an approach can neither directly estimate the generative mechanism of spike trains nor use stimulus information that has a strong influence on neural activities. Here, we propose a new deconvolution method for calcium imaging using marked point processes. We consider that the observed movie is generated from a probabilistic model with marked point processes as hidden variables, and we calculate the posterior of these variables using a variational inference approach. Our method can simultaneously estimate various kinds of information, such as cell shape, spike occurrence time, and tuning curve. We apply our method to simulated and experimental data to verify its performance.

## Author summary

Calcium imaging is a promising technique that enables the observation of the activities of large neural populations as a movie. Since the measured movie is a large-scale dataset containing a considerable amount of information, we need to apply a preprocessing procedure to extract crucial information from the raw movie for the analysis that follows. Recent studies have adopted matrix decomposition to decompose the observed movie into the product of two matrices: one consisting of cell shapes and the other consisting of calcium florescent time series. This approach can estimate cell locations and activities simultaneously; however, it cannot express some aspects of neural population codes. For instance, this approach cannot incorporate other covariates that may affect the neural population activities. In this paper, we propose a new statistical model for calcium imaging movies and an estimation procedure for this model. To express the random occurrence of spikes occurring in the movie, our model adopts a marked point process, which is used to express sequences of events to which certain characteristic values are attached. Our model can estimate cell shapes, spikes, and tuning curves of cells directly without any additional preprocessing procedure, and it also improves the estimation accuracy compared to the conventional approach.

This is a *PLOS Computational Biology* Methods paper.

## Introduction

In recent years, many researchers have used calcium imaging to measure spiking activities in large neuron populations. Calcium imaging is an imaging technique that observes calcium ion concentrations inside neurons as a movie. This technique enables researchers to obtain various types of information about the neurons that cannot be obtained by the potential measurement approach.

Although calcium imaging offers a considerable amount of information, it is difficult to deal with the observed movie directly. Therefore, when using calcium imaging to investigate the structure of the brain system, we need to extract crucial information from the raw movie beforehand. In calcium imaging, for example, the cell shapes, positions, and spiking times of neurons are extracted from the raw movie.

**Fig 1:**
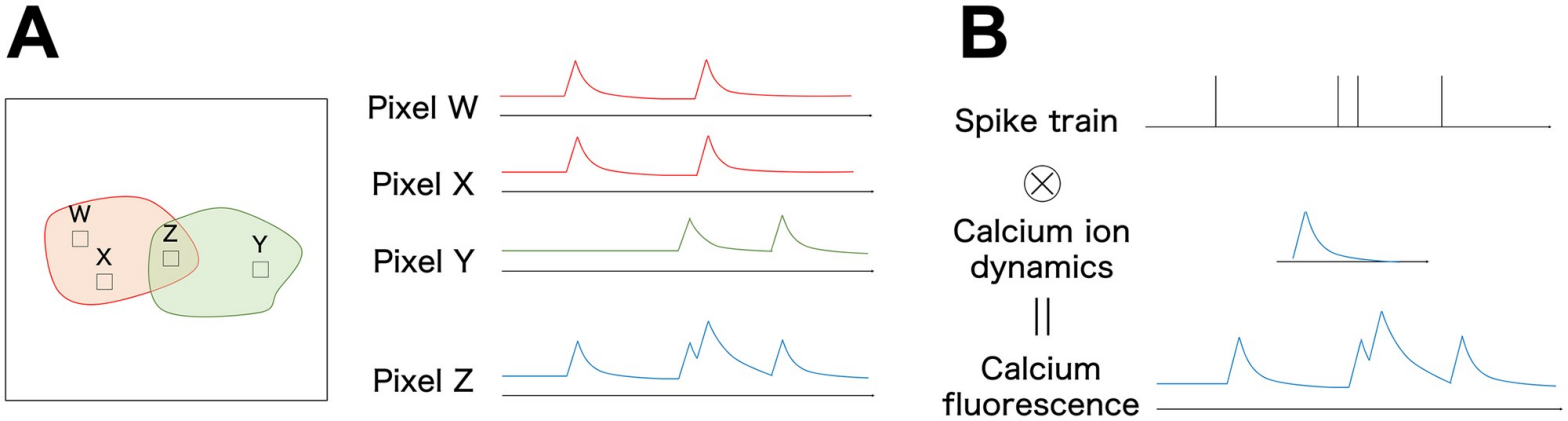
Characteristic features of a calcium imaging movie. (A) Cell overlap in the spatial domain. Calcium fluorescence observed at pixels W and X contained in the same neuron shows similar behavior, which differs from the calcium fluorescence observed at pixel Y contained in another neuron. The calcium fluorescence observed at pixel Z is the sum of these series when pixel Z is contained by both neurons. (B) Calcium fluorescence overlap in the temporal domain. Calcium fluorescence observed at each pixel is the convolution of calcium ion dynamics into a spike train. If two spikes occur in a short time relative to the time constant of these dynamics, increases in calcium ion concentration due to these spikes will overlap each other.

Two statistical problems arise with this preprocessing. The first problem involves detecting the shape of each neuron. When a neuron spikes, the fluorescent value at the pixel contained within this neuron rises sharply. Therefore, to ascertain the firing activity of each neuron, it is necessary to know which pixels belong to a particular neuron. Some previous studies have calculated the summarized image averaged in the time direction and applied an image segmentation method to clarify the locations of the neurons [1, 2]. However, each pixel in the movie may be contained in two or more neurons, and the observed calcium ion concentrations at such pixels become the sum of the calcium concentrations for the two neurons. Fig 1A illustrates such overlaps in the spatial domain. If neurons overlap each other, we need to deconvolve such overlaps to extract the calcium fluorescent dynamics of each neuron.

The second problem involves detecting the spike occurrence time. Calcium concentration is observed as a continuous time series that sharply increases when the neuron fires and subsequently slowly decays exponentially with a small time constant. If multiple spikes occur within a short time, slow decay of calcium fluorescence may cause these firings to overlap in the temporal domain in the movie. Fig 1B illustrates the overlap in the temporal domain. Therefore, to extract a spike train from the movie, we must also deconvolve calcium traces in the temporal domain. Previous studies provide several types of solutions for such deconvolution problems [3, 4, 5, 6]. Deconvolution methods for a one-dimensional calcium transient require prior assignment of pixels to neurons to calculate the one-dimensional mean fluorescent transient of each neuron. For a review for these methods, see [7].

To solve these two problems simultaneously, recent studies have proposed a matrix deconvolution approach [8, 9, 10]. In this approach, the observed movie is converted into a matrix form, and this matrix is approximated by the product of two different matrices: one contains the cell shapes in its columns and the other contains the calcium fluorescent series in its rows. Initially, [8] proposed a deconvolution method using independent component analysis and succeeded in deconvolving a calcium imaging movie in both the temporal domain and the spatial domain simultaneously. However, because independent components analysis is a linear decomposition, it cannot deal with the nonlinearity of a calcium imaging movie. To address such nonlinearity, [9] introduced nonnegative matrix factorization to this deconvolution. Nonnegative matrix factorization can perform nonlinear decomposition, thereby allowing the effective separation of neuron overlapping. Recently, [10] used constrained nonnegative matrix factorization to incorporate the nature of calcium ion dynamics.

The matrix deconvolution approach deconvolves the calcium imaging movie simultaneously in both the temporal and spatial domains, and it can dramatically improve the deconvolution accuracy. However, such an approach still suffers from several problems. First, it cannot detect spike trains as 0–1 sequences. Using matrix deconvolution, we solve the optimization problem to obtain two matrices whose product approximates the movie matrix well. In the optimization step, we relax the 0–1 condition imposed on the time series expressing spiking activities. Consequently, to estimate spike occurrence time from the deconvolution results, we must perform an additional threshold procedure, which may cause either overestimation or underestimation of the number of spikes. Second, even if there are covariates that may influence spiking activities, we cannot incorporate this information in the deconvolution procedure. This affects the estimation of the tuning curve, which is the function of the firing rate with respect to an external stimulus.

Separating spike assignment and subsequent estimation as distinct steps may cause errors and loss of information. This problem has also been reported in neural decoding research. When applying neural decoding, we need to create spike trains from the measured potential beforehand by using a preprocessing procedure called spike sorting. Spike sorting assigns spikes to neurons by applying clustering methods to the waveform information of spikes. This preprocessing generally does not use stimulus information in this spike assignment, and it causes bias and a loss of information for tuning curve estimation [11]. To avoid such information loss, clusterless decoding methods using marked point processes are proposed [12, 13, 14]. In this approach, spike sequences and the attached waveform information are expressed as marked sequences, and the distribution of the marked sequences is directly estimated. Clusterless decoding enables us to avoid performing spike sorting and improves the decoding accuracy.

Such a problem due to two-step analysis also occurs in calcium demixing. Even if the aim is to investigate the relation between spike occurrence and other simultaneously measured covariates, the existing approaches ignore this covariate information, which may be most effective for extracting spiking activities from the raw movie. Consider two overlapping neurons that are tuned to an external stimulus, and their receptive fields are isolated. Then, spikes emitted from these two neurons can be easily assigned to true components by observing the stimulus value at each spike occurrence time. However, the existing approaches do not use this relation, resulting in the misassignment of the spikes. Moreover, existing approaches assume that the spikes occur at a constant firing rate along the time axis even though the analysis that follows is based on the existence of the tuning curves. Such model misspecification leads to bias in detecting the spikes and cell shapes in the demixing phase, and this bias also affects the subsequent analysis. Therefore, the simultaneous extraction of the spikes and estimation of the tuning curves has various advantages in calcium imaging analysis as well.

In this paper, we propose a new deconvolution method using a probabilistic generative model. We consider that the calcium imaging movie is generated from a stochastic process that has marked point processes as hidden variables and obtains various types of information as a posterior of the hidden variables using variational Bayesian inference. The marked point process is a stochastic process used to express a random series of events with characteristic values called marks. We express the injected calcium added to the pixels in the imaging movie when a spike occurs as vectors, and call these vectors as marks. Our model expresses the distribution of the sequences of the marks as the marked point process. Reflecting the dependent structure between the spiking activities and the stimulus change in the definition of the marked point processes, we simultaneously perform calcium demixing and tuning curve estimation. This generative modeling avoids a two-step analysis and reduces the error and loss of information that occurred in previous approaches.

The remainder of this paper is organized as follows: In Results, we first apply our method to the simulated data and verify its performance. We also apply our method to a measured calcium imaging movie to extract the place cell spiking activities. In Discussion, we discuss our method. We provide the details of our generative model and the estimation procedure in Methods and S1 Appendix.

## Results

### Simulation study

In this section, we apply our method and the existing approach to artificially generated data and compare their performance. The details of the mathematical terms that appear in this section are explained in Methods.

First, we generated the data using a model. Consider that the activities of nine neurons are observed simultaneously by calcium imaging and that each neuron emits spikes corresponding to external stimulus $x_{t}\in R$. Let *κ* be a vectorization of the fluorescence image, and assume that the marked spike sequence of the *k*-th neuron is generated from the marked inhomogeneous Poisson process modulated by *x*_*t*_ whose intensity is

$$\begin{array}{c} \lambda_{k}\;N\left(\kappa |\mu_{k}^{\kappa },\Lambda_{k}^{\kappa }\right)\;N\left(x|\mu_{k}^{x},\tau_{k}^{x}\right). \end{array}$$

Here, N(⋅∣⋅) is a Gaussian kernel, λ_*k*_ is a mean rate, $\mu_{k}^{\kappa }$ is the mean vector of the density that explains the fluorescent image fluctuation of this neuron, $\mu_{k}^{x}$ is a stimulus value to which this neuron reacts most extremely, and $\Lambda_{k}^{\kappa },\;\tau_{k}^{x}$ are the scale parameters for *κ* and *x*, respectively. Then, we assumed that the spike train of each neuron is generated independently, given *x*_*t*_. This means that the overall intensity function is expressed as

$$\begin{array}{c} \sum_{k=1}^{9}\lambda_{k}\;N\left(\kappa |\mu_{k}^{\kappa },\Lambda_{k}^{\kappa }\right)\;N\left(x|\mu_{k}^{x},\tau_{k}^{x}\right). \end{array}$$

**Fig 2:**
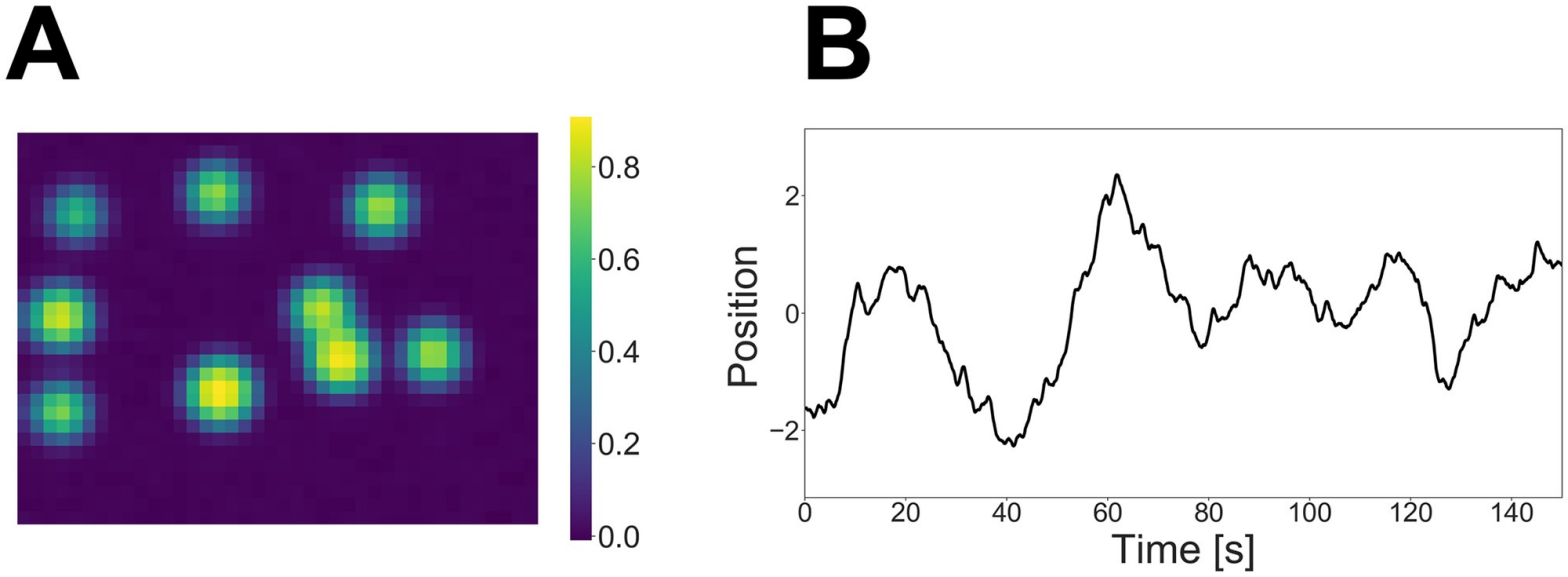
(A) Correlation image calculated from the simulated movie. (B) External stimulus used for data generation.

Under such an assumption, we generated a marked spike sequence from this marked point process. Given this sequence, we generated a movie that lasted for 150 s including jumps from the state space model, which is defined as (1) and (2). We omit the details of the other hyperparameters’ settings in this paper. A correlation image calculated from the generated movie and the external stimulus used for the data generation are shown in Fig 2. Each pixel of the correlation image contains correlation coefficients with neighboring pixels. It indicates which parts of the movie change simultaneously so that we can roughly estimate the spatial locations of the neurons from it.

Next, we applied our method to the generated movie. For kernel function *f*^*x*^(*x* ∣ *θ*^*x*^) that expresses the tuning curve of each cell, we selected the univariate Gaussian kernel

$$\begin{array}{c} f^{x}\left(x|\mu^{x},\tau^{x}\right)=N\left(x|\mu^{x},\tau^{x}\right) \end{array}$$

and set the dimension of hidden state *d*^*κ*^ as 40 and the number of mixtures *K* as 20. In the estimation step, we initialized the distribution of the hidden variables and the hyperparameters, as described in S1 Appendix. Then, we updated these distributions and hyperparameters for 10 iterations and obtained the variational posterior $\hat{q}$. Using the calculated variational posterior, we found the variational expectation of the hidden variables to obtain the deconvolution results.

To compare the performance of our method with the existing approach, we also applied constrained nonnegative matrix factorization proposed by [10]. Hereafter, we denote this method as CNMF. Since CNMF estimates the spike sequence as the real value sequence and not as the 0–1 sequence, we decided a threshold value for each neuron and detected the spike occurrence time from these estimated spike sequences using this threshold. Specifically, we calculated distances *d*_*r*_, *r* = 1, …, *R* from the estimated spike sequences $s_{r}\in R,\;r=1,\ldots ,R$ as

$$\begin{array}{c} d_{r}=\sqrt{\frac{{\left(s_{r}-\tilde{\mu }\right)}^{2}}{\tilde{\sigma^{2}}}}, \end{array}$$

where $\tilde{\mu }$ is a sample mean and $\tilde{\sigma }$ is a sample variance calculated from *s*_*r*_. Using these distances, we considered that spike occurred at *r* if $d_{r}^{2}$ is larger than the upper 0.1% point of the chi-squared distribution with one degree of freedom. Then, we assumed that the detected spike train of the *k*-th component is generated from the unmarked inhomogeneous Poisson process modulated by *x*_*t*_. Under such a Poisson assumption, the intensity function can be expressed as a function of *x*_*t*_; this function is called the tuning curve in neuroscience. We assumed that the tuning curve of the *k*-th component is expressed as

$$\begin{array}{c} \lambda \left(x|\mu_{k}^{x},\tau_{k}^{x}\right)=\lambda_{k}\;N\left(x|\mu_{k}^{x},\tau_{k}^{x}\right) \end{array}$$

and estimated the parameters $(\lambda_{k},\mu_{k}^{x},\tau_{k}^{x}),\;k=1,\ldots ,K$ using the maximum likelihood estimation. For details of the tuning curve estimation, see [15], for example.

**Fig 3:**
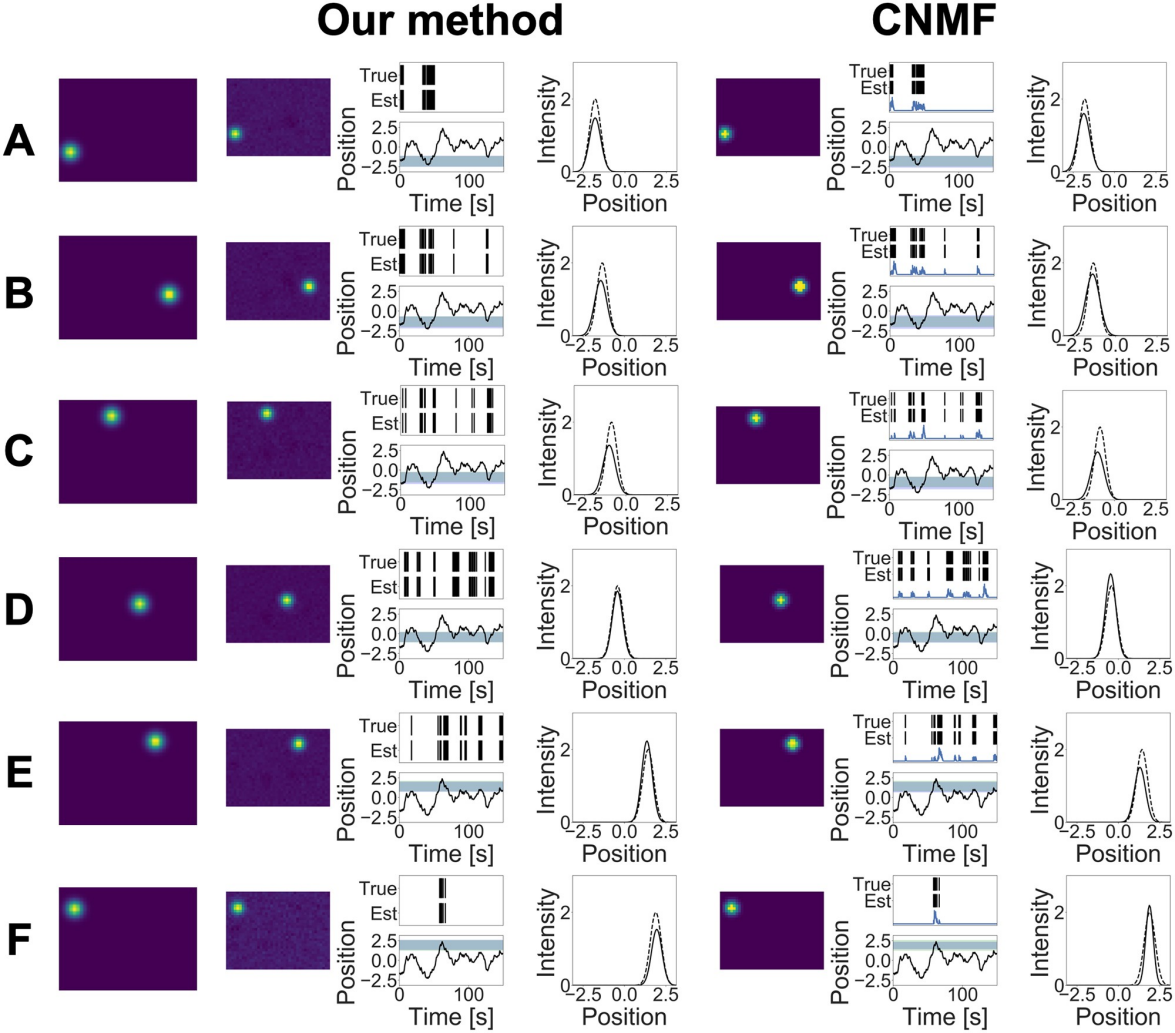
(A)–(F) Estimation results obtained by two methods in the simulation study. The first column indicates the ground truths, the second column shows components obtained by our method, and the third column shows components obtained by CNMF. Each component consists of three figures. (Left) Estimated cell shape. (Middle) Estimated spike train. The vertical lines indicate when spikes occurred; the upper lines indicate true spikes, and the bottom lines indicate estimated spikes. The black line plot shows the external stimulus. The green band shows the receptive field decided by the true *μ*^*x*^ and *τ*^*x*^, and the blue band shows the receptive field decided by the estimated values. (Right) Tuning curve. The solid line shows the estimated tuning curve, and the dashed line shows the true tuning curve with the mean value closest to the estimated mean value $\mu_{k}^{x}$.

**Fig 4:**
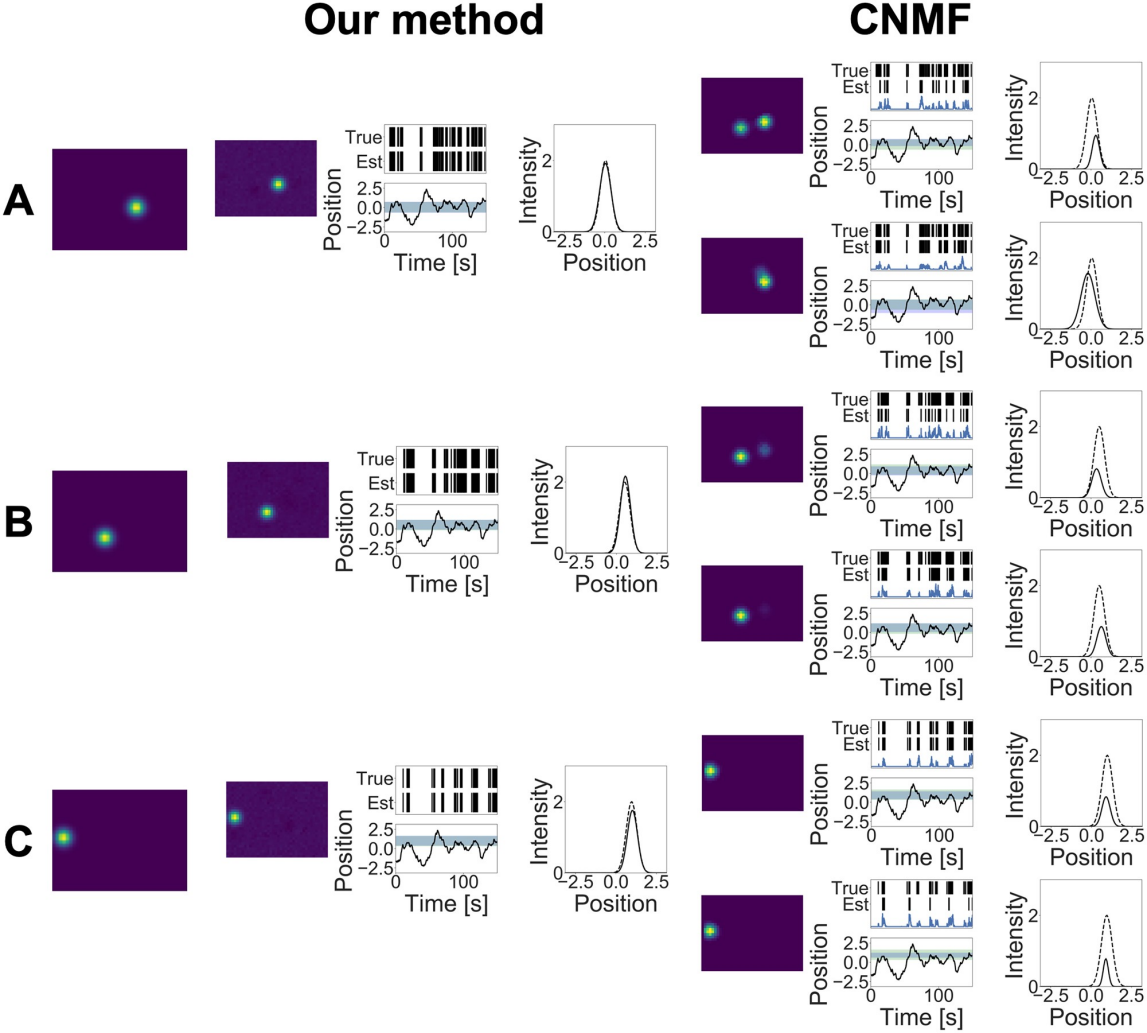
(A)–(C) Estimation results obtained by two methods in the simulation study.

The estimation results of our method and CNMF are shown in Figs 3 and 4. Our method detected 9 neurons while CNMF detected 12 neurons, and this result shows that our method was able to estimate the true number of neurons, whereas CNMF overestimated this number. Although CNMF contains heuristic procedures such as merging and discarding components, to detect the number of neurons, it cannot adjust the model complexity along the minimization of their loss function. By contrast, our method uses the weighted gamma process as a prior and can perform model selection automatically in the estimation step.

Figs 3 and 4 also show that our method estimated the cell shape and the tuning curve of each neuron accurately. Conversely, CNMF underestimated the tuning curve for some neurons. Adjusting threshold values may reduce such estimation errors; however, it is difficult to determine these values properly beforehand. Specifically, components 4A, 4B, and 4C in Fig 4 are separated into two different components in CNMF. If CNMF can incorporate the covariate information into calcium demixing, then it would integrate these components into one component by observing similar tuning profiles. This result shows the advantage of using the tuning curve at demixing phase.

To compare the performance quantitatively in terms of spike detection and regions of interest (ROI) detection, we calculated the *F*-measure to evaluate the performance of binary classification. It is defined as the harmonic mean of precision and recall:

$$\begin{array}{c} F_{\beta }=\frac{(1+\beta^{2})\;\text{precision}\cdot \text{recall}}{\beta^{2}\;\text{precision}+\text{recall}}. \end{array}$$

The mean values of the *F*-measures in terms of spike detection were 0.998 (our model) and 0.808 (CNMF) with *β*^2^ = 0.3. On the other hand, the mean values of the *F*-measures in terms of ROI detection were 0.985 (our model) and 0.503 (CNMF) with the same *β*^2^ value. These values show that our model can detect both spike times and cell shapes better than CNMF.

We also compared the two methods in terms of tuning curve estimation. In this comparison, we ignored the marks and assumed that the spike train was generated from one neural population modulated by *x*_*t*_. Here, the tuning curve of this neuron population corresponds to the summation of all estimated tuning curves. Under this assumption, we calculated the likelihood, given the ground truth spikes. Then, the log likelihood of our model and CNMF was 92.88 and 85.16, respectively, indicating that our model provided better tuning curve estimates than CNMF.

### Application to the experimental dataset

In this section, we apply our method to the dataset published by [16, 17, 18]. The authors investigated place cell dynamics of hippocampus CA1 between wild-type and Df(16)*A*^+/−^ mice, an animal model of the 22q11.2 deletion syndrome, by using two-photon Ca^2+^ imaging with GCaMP6f. In their experiment, mice on a treadmill were imposed to a head-fixed goal oriented task. The treadmill was composed of a 2 m-long belt consisting of three different fabrics and six different tactile cues. The mice searched for hidden rewards on the treadmill using these cues as clues. After the recording, the authors obtained the ROI from the observed movie by drawing GCaMP6f-labeled somata manually. The number of detected cells per wild-type mouse was 463 ± 37 (mean ± std, *n* = 6).

One imaged session of a wild-type mouse is used in our paper. Its frame rate was 7.5 Hz. The length of the movie was 2250 frames, the size of the one frame was 498 × 490 pixels, and there were 1.7007 pixels per micron. For the experimental details, see [17].

**Fig 5:**
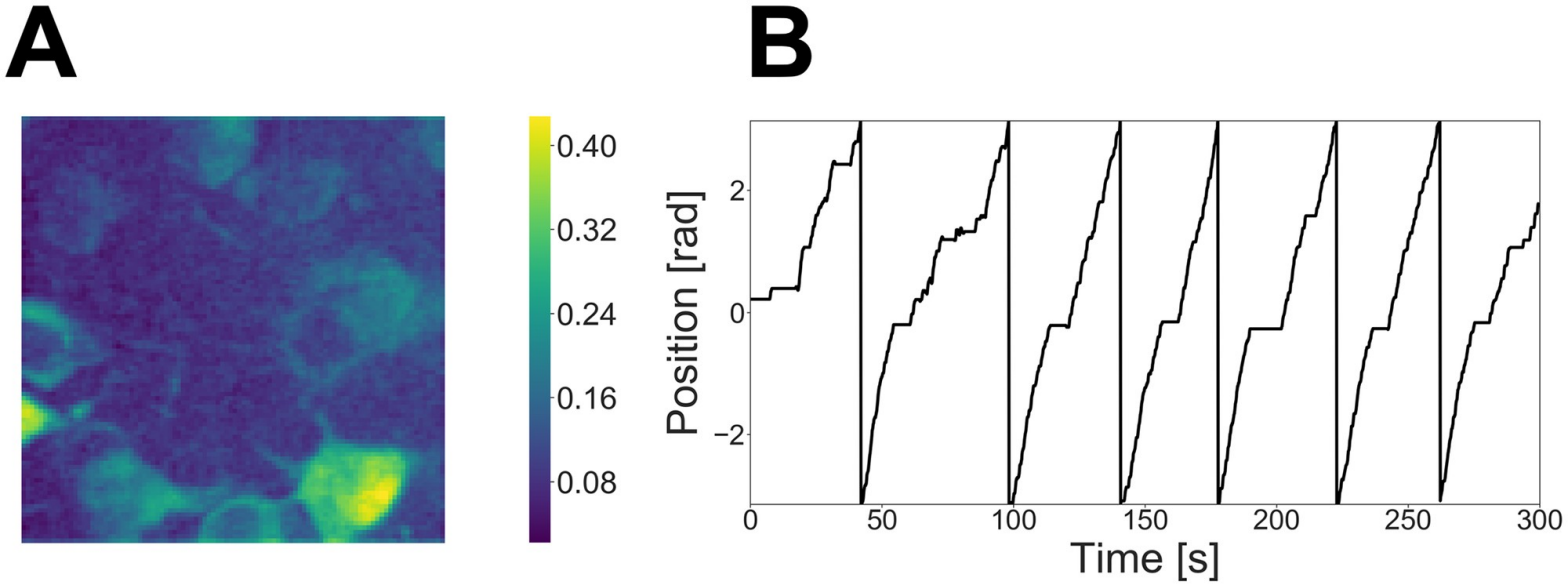
(A) Correlation image calculated from the calcium imaging movie. (B) Mouse trajectory throughout the experiment.

The size of the movie in this dataset was approximately 500 × 500, and it was too large for our method. Therefore, we divided the raw movie into 25 patches, each sized approximately 100 × 100 (*d*^*y*^ = 10000), and applied our method to each patch independently. In this section, we show the result for one of these patches. A correlation image calculated from this patch and the mouse’s position on the treadmill during the task are shown in Fig 5.

A place cell has a place field, and it fires when the animal passes through its place field [19]. Hence, we assumed that the position of the mouse on the treadmill is external stimulus *x*_*t*_ and that it affects the spiking activities of the neurons. We regarded this treadmill as a unit circle in $R^{2}$ and defined the external stimulus *x*_*t*_ by the angle of this circle with range (−*π*, *π*). For the kernel function *f*^*x*^(*x* ∣ *θ*^*x*^), we selected the von Mises kernel

$$\begin{array}{c} f^{x}\left(x|\theta^{x}\right)=\text{VM}\left(x|\mu^{x},\tau^{x}\right), \end{array}$$

where *μ*^*x*^ and *τ*^*x*^ are the center location and the scale parameter, respectively, of its place field. We also set *d*^*κ*^ = 40 and *K* = 30.

In the estimation step, we initialized the distribution of the hidden variables and the hyperparameters as described in S1 Appendix. Then, we updated the distribution and the hyperparameters for 10 iterations and obtained the variational posterior $\hat{q}$. Using this variational posterior $\hat{q}$, we calculated the variational expectation of hidden variables to obtain the deconvolution results.

To compare performance, we also applied CNMF proposed by [10]. Similar to Simulation study, we decided threshold values to detect spikes from the deconvolution results of CNMF. Then, we assumed that the detected spike train of the *k*-th component is generated from an unmarked point process whose intensity is

$$\begin{array}{c} \lambda \left(x|\theta_{k}^{x}\right)=\lambda_{k}\;\text{VM}\left(x|\mu_{k}^{x},\tau_{k}^{x}\right) \end{array}$$

and estimated the parameters $(\lambda_{k},\mu_{k}^{x},\tau_{k}^{x}),\;k=1,\ldots ,K$ using maximum likelihood estimation.

**Fig 6:**
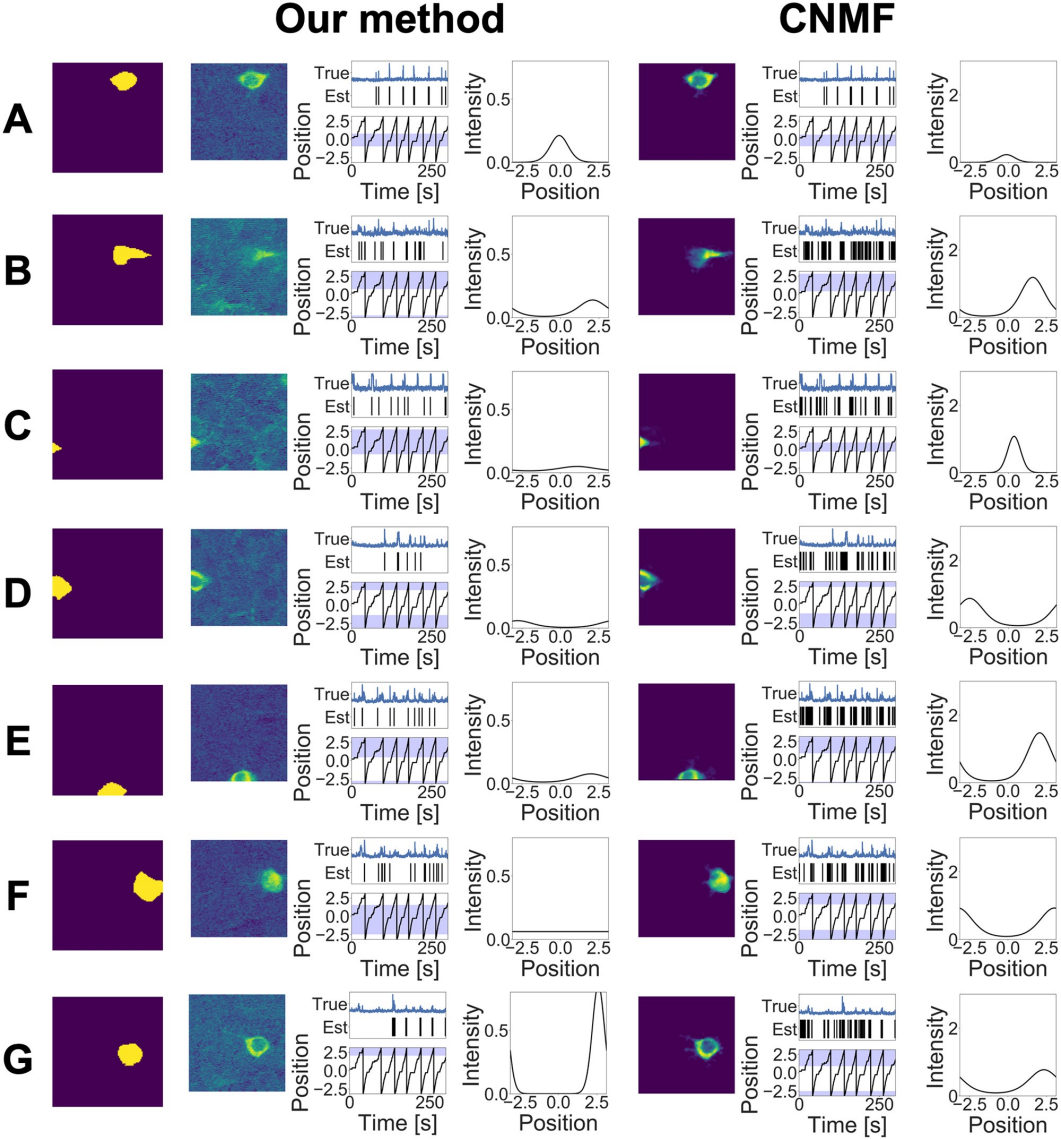
(A)–(G) Estimation results obtained by the two methods in the experimental study. The first column indicates the ground truths, the second column shows the components obtained by our method, and third column shows those obtained by CNMF. Each component consists of three images. (Left) Estimated cell shape. (Middle) Calcium trace, estimated spiking time, and receptive field. The blue line indicates the normalized calcium trace within the ROI, and each vertical line indicates the estimated index when the spike occurred. The black line plot shows the position of the mouse, and the blue band shows the receptive field decided by *μ*^*x*^ and *τ*^*x*^. (Right) Estimated tuning curve.

**Fig 7:**
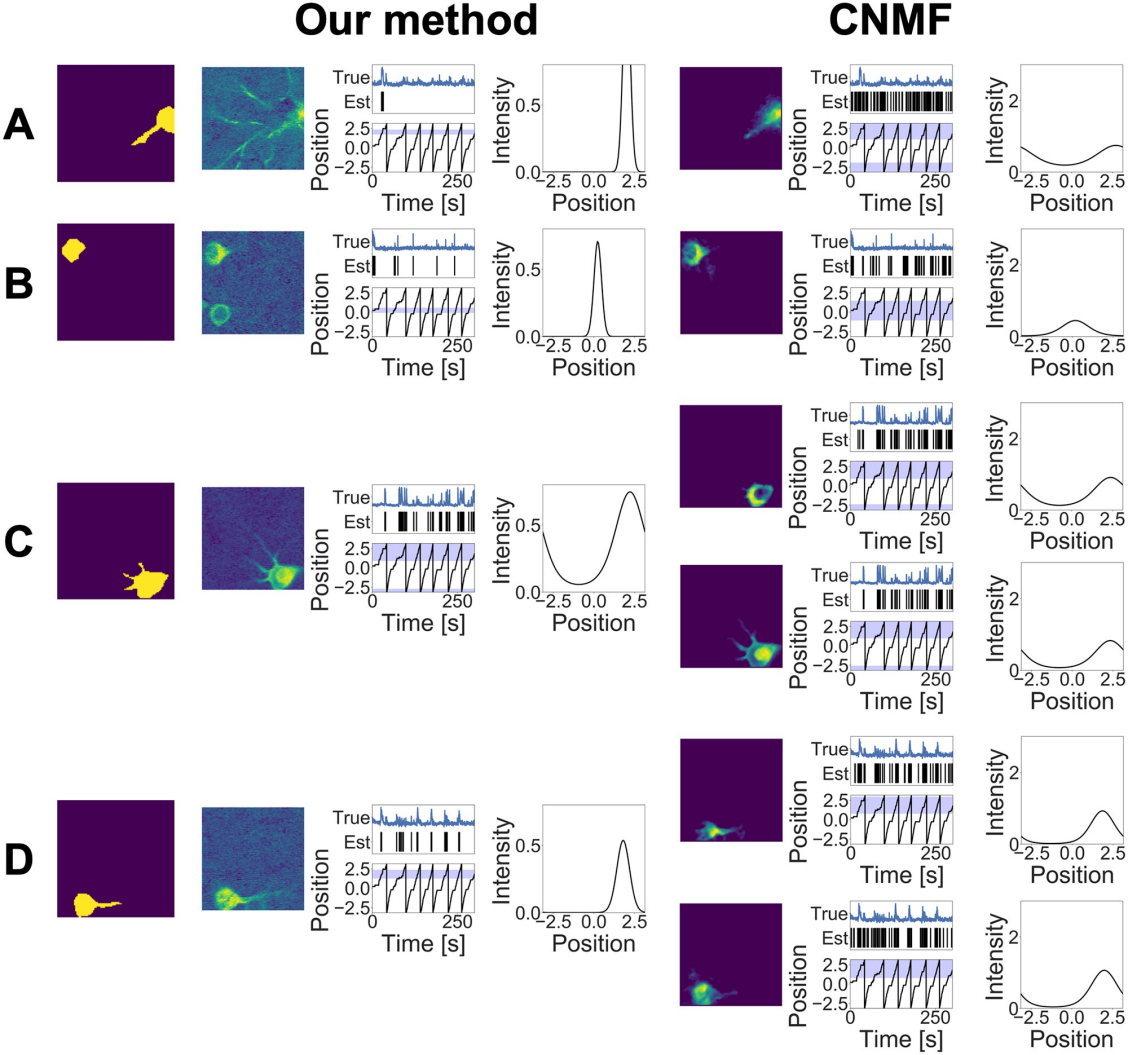
(A)–(D) Estimation results obtained by the two methods in the experimental study.

**Fig 8:**
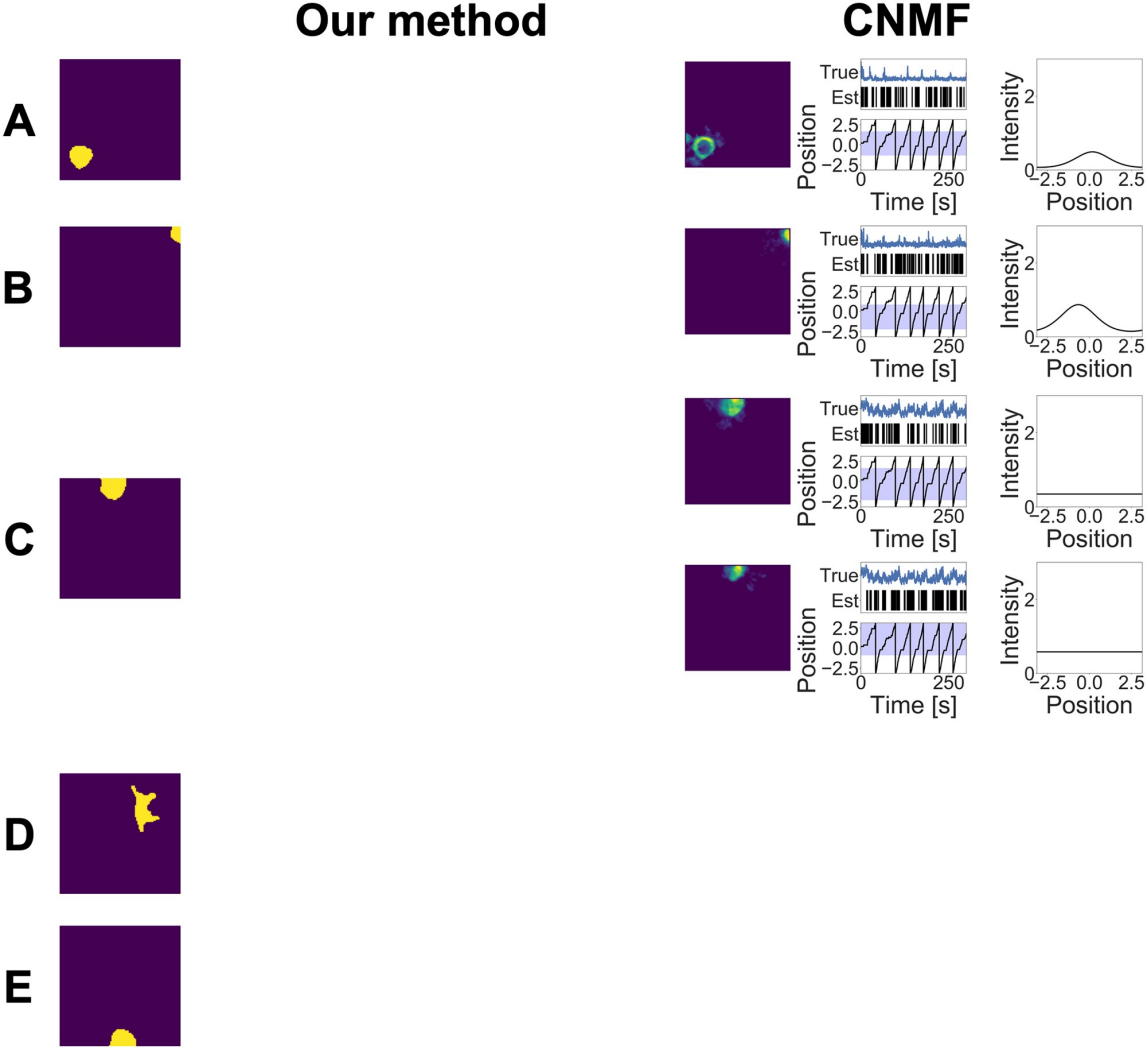
(A)–(E) Estimation results obtained by the two methods in the experimental study.

The estimation results of our method and CNMF are shown in Figs 6, 7 and 8. In order to evaluate our model performance in a quantitative manner, we manually detected the ROI of the neurons located in the raw movie. These ground truths of the ROIs and the corresponding calcium traces calculated by these ROIs are also shown in these figures. Note that the calcium traces shown in these figures are equal to the mean calcium values within each ROI and they do not necessarily reflect true calcium traces; they contain calcium fluctuations arising from other overlapping neurons and background noise. Our method detected 11 components and CNMF detected 17 components. From this point on, we denote the estimated component whose label is (A) in Fig 6 as Neuron 6A.

Fig 6 shows that the spike trains obtained by our method and the calcium fluorescence obtained by CNMF are consistent for the neurons whose cell shapes are similar. For example, the spikes estimated by both methods for Neuron 6A are almost the same. This observation shows that our method provides estimations consistent with the existing approach.

Next, we compare each component estimated by the two methods in detail. Initially, we focus on the difference in terms of the estimated cell shapes between our method and CNMF. The estimated cell shapes for Neuron 7A are totally different. This is due to the regularization constraints imposed in CNMF. Because CNMF imposes regularization constraints to the estimated cell shape as being localized, it may fail to estimate neurons that are widely spread. As our method does not impose such a requirement, it can detect neurons whose cell shape is widely spread on the microscope plane.

The cell shape estimated by our method for Neuron 7B contained Neuron 8A, whereas CNMF correctly divided them into two components. This is because our model does not use any spatial information; the cell has a localized shape and is not divided into two or more parts separately. In CNMF, the initial value for the shape of the neuron is defined as being localized. Therefore, two neurons are regarded as different components as long as the their positions are isolated. We can introduce such spatial assumptions into our model as regularization or heuristics; however, it may result in miss-detection for a wide spread neurons. For instance, if we define the limit size of the cell shape beforehand, we may fail to detect components such as Neuron 7A. If Neurons 7B and 8A have different tunings to the stimulus, our method would be able to distinguish them. However, these neurons fired simultaneously and had similar tuning to the covariates within the observation interval. The other reason for the result is that these neurons had similar calcium fluorescence. This observation indicates that these neurons may share another relation behind and should not be separated.

Neuron 7C, estimated as a single neuron by our method, was divided into two components in CNMF, and Neuron 7D was also divided into two components. Such differences arise from the definitions of the calcium footprint in the two methods. Our model assumes the calcium footprint to be a random variable and thus allows random fluctuations to a certain extent. On the other hand, CNMF expresses the footprints as deterministic vectors and divides the random fluctuation of one neuron activity into two or more components. The assumption of the tuning curve existences in our model also works as a regularization for merging components with similar tuning profiles into one component.

Neurons 8B and 8C detected by CNMF were not detected by our method. These components had a common feature: the estimated calcium fluorescences of these components were noisy. Because of the high fluctuation of the calcium fluorescence, the number of spikes of these components was larger than that of the other components, which seems to be unreliable. Our method discards components whose calcium fluorescence cannot be approximated by convolution exponential decay with the spike sequence.

Neurons 8D and 8E were not detected by both methods. This is because the activities of these neurons were too small within the observed interval. Both methods considered these components as background noise fluctuation.

To compare the performance in terms of ROI detection, we calculated the *F*-measures of the two methods. We first detected the ROI of each neuron manually from the correlation image shown in Fig 5. The mean values of the *F*-measures are 0.578 (our model) and 0.501 (CNMF) with *β*^2^ = 0.3. This result also shows the advantage of not using spatial information in our model.

Next, we compare the results in terms of spike detection and tuning curve estimation. On the whole, the tuning curves estimated by our method are sharper than those estimated by CNMF. This difference is due to the assumption of tuning curve existence at the demixing phase. CNMF assumes that the spikes are generated at a constant firing rate along the time axis, and thus, it tends to express the background fluctuation in the interval where true spikes do not occur as false positive spikes. For example, for Neuron 7D, the estimated spikes and tuning curves of the two methods are different. The calcium traces obtained using ground truth increase when the mouse passes through a specific area, and our method can express this response of the neuron to the mouse’s movement. On the other hand, CNMF treats background fluctuations as spikes and estimates the tuning curve as a more widely spread shape.

## Discussion

When using calcium imaging to measure the spiking activities of neurons, we need to extract the summarized information from the raw movie in advance. Recent studies have used the matrix deconvolution approach for this preprocessing; however, such an approach ignores the spiking nature of the neurons, and it cannot use the covariates information, which may have a great influence on the spiking activities.

To solve such problems, we proposed a new deconvolution method using a probabilistic approach. In our method, we assume that a calcium imaging movie is generated from a generative model with marked point processes as hidden variables, and we calculate the posterior using a variational inference approach. Our method detects cell shapes and spike times, and estimates tuning curves simultaneously, which reduces estimation biases and loss of information that occurs in the conventional two-step analysis. By applying our method to simulated and experimental data, we showed that it can estimate various types of information simultaneously from a raw movie.

Although our method provides a new framework for calcium fluorescence deconvolution, several problems exist at this point. Hereafter, we discuss the disadvantages of our method and suggest future extensions of this work.

Our model assumes that spike trains of different neurons occur independently, given external covariates. However, this assumption is quite strong; cortical spike trains generated by different neurons have some positive or negative correlations. Our model ignores such correlations as introducing such structure into the generative model makes estimations difficult. We aim to improve the model via the inclusion of such correlations in the future.

Our method approximates the true posterior using variational inference, and the variational posterior obtained by our method may differ from the true posterior. If this difference is not negligible, the estimation results may be unreliable. To obtain a good solution, we must relax the independence requirement imposed on the variational posterior. It is also important to investigate the asymptotic behavior of our method to evaluate the difference between the true posterior and the variational posterior.

Our method can be applied to various kinds of analyses of calcium imaging movies. One example is neural decoding. Decoding an external stimulus from a calcium imaging movie has already been attempted in previous studies [20]. In these studies, spike trains are detected from the calcium imaging movie beforehand using preprocessing procedures, and decoding is treated as another step in this preprocessing. As mentioned in Introduction, separating spike assignment and subsequent estimation as distinct steps causes errors and loss of information. On the other hand, since our method assigns spike trains and estimates the tuning curve simultaneously, it may be able to improve the decoding accuracy. Moreover, our method will enable us to decode stimuli from a calcium imaging movie in an online manner because it provides the probability distribution for the calcium imaging movie itself and does not require preprocessing beforehand.

## Methods

We focus on neuron populations whose firing rates change according to an external stimulus and on the measurement of these neurons by calcium imaging. The explanation for our method comprises two parts. First, we explain a probabilistic model that decides the generative mechanism of the calcium imaging movie. Next, we demonstrate the method for estimating the hidden variables of this generative model.

### Generative model

Our model comprises three components. The first component explains how calcium fluorescent dynamics construct the observed movie, given the spiking activities of neurons. The second component describes how these spiking activities are generated depending on the external stimulus. The third component specifies a prior for the parameters used in the first and second components.

Box 1 shows the summary of our generative model in mathematical form. The definitions of the distributions and kernels we use in our generative model are also summarized in S1 Appendix. To distinguish the distributions and probability density functions, we express the density functions as shorthand notations.

**Box 1: Summary of our generative model**

- Calcium fluorescence model
  $$\begin{array}{c} c_{1}∼\text{Normal}\left(\mu_{\text{init}}^{c},{\left(\Sigma_{\text{init}}^{c}\right)}^{-1}\right),\\ \left\{ \begin{array}{cc} c_{r}=Fc_{r-1}+\kappa_{r}, & \text{(if}\;\kappa_{r}\neq \emptyset \text{),}\\ c_{r}=Fc_{r-1}+v_{r},\;v_{r}∼\text{Normal}\left(0,V\right), & \text{(if}\;\kappa_{r}=\emptyset \text{),} \end{array}\right.\;r=2,\ldots ,R,\\ y_{r}=Gc_{r}+w_{r},\;w_{r}∼\text{Normal}\left(0,W\right),\;r=1,\ldots ,R, \end{array}$$
- Spike generation model
  $$\kappa ∼\text{MarkedPointProcess}(\lambda (\kappa |x_{t},\xi ))$$
  $$\begin{array}{cc} \lambda (\kappa |x_{t},\xi ) & =\int_{\Theta }f\left(\kappa ,x_{t}|\theta \right)\xi \left(d\theta \right)\\ & =\int_{\Theta^{x}}\int_{\Theta^{\kappa }}f^{\kappa }\left(\kappa |\theta^{\kappa }\right)\;f^{x}\left(x|\theta^{x}\right)\;\xi \left(d\theta^{\kappa },d\theta^{x}\right). \end{array}$$
- Prior
  $$\begin{array}{c} \xi ∼G_{K}(d\xi |\alpha_{0},\beta_{0},H^{\kappa },u^{x}). \end{array}$$
- Other variables
  $$\begin{array}{cc} & x\;\text{is}\;\text{given,}\\ & (\mu_{\text{init}}^{c},\Sigma_{\text{init}}^{c},F,V,G,W,\alpha_{0},u^{x})\;\text{are}\;\text{hyperparameters}\;\text{to}\;\text{be}\;\text{estimated,}\\ & (\beta_{0},m^{\kappa },\gamma^{\kappa },\nu^{\kappa },S^{\kappa })\;\text{are}\;\text{fixed}\;\text{hyperparameters.} \end{array}$$

#### Calcium fluorescence model

**Fig 9:**
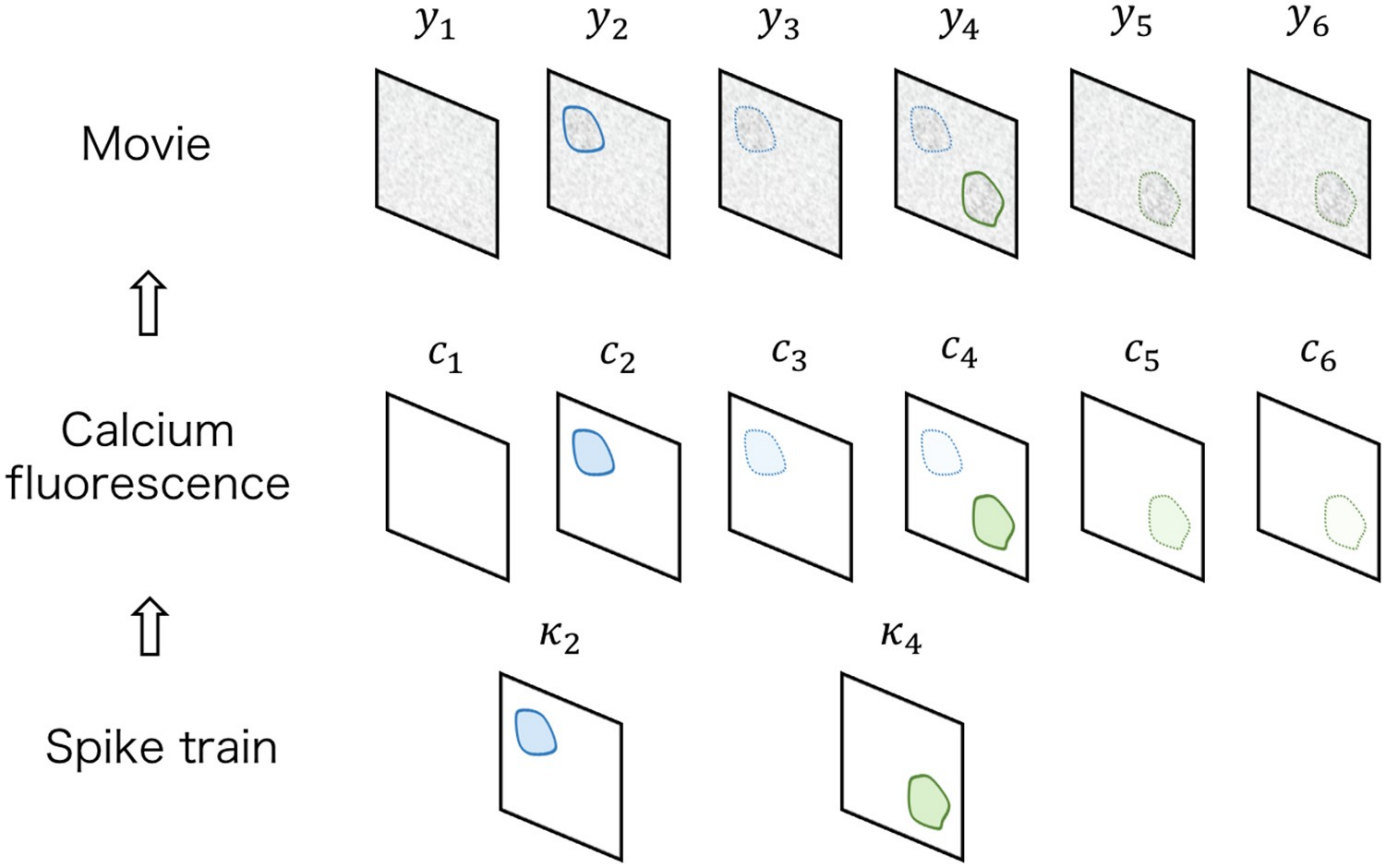
Calcium fluorescence model. The marked spike sequences ***κ*** contain fluorescent footprints of the spikes. Given ***κ***, the calcium fluorescence ***c*** and the observed movie ***y*** are generated from a linear state space model including jumps. Because *c*_*r*_ and *κ*_*r*_ are not defined on the space of *y*_*r*_, these variables are represented as transformed versions *Gc*_*r*_, *Gκ*_*r*_ in this figure for intuition. When a spike occurs, the footprint of this spike is added to the calcium fluorescence, and then, it gradually decreases according to the calcium ion decay dynamics. The blue and green components indicate that the fluorescent footprints of the spikes originated from different neurons.

Our generative model assumes that there is a hidden stochastic process that explains the dynamics of the true calcium fluorescence and that the observed movie is the linear transformation of this process. Fig 9 illustrates this component.

We define several variables. Let (0, *T*] be an observation interval, and consider that the calcium imaging movie is measured in this observation interval at sampling interval Δ. Assume $R=\frac{T}{\Delta }$. Let $c_{r}\in R^{d^{\kappa }},\;r=1,\ldots ,R$ be the values of this hidden process and $y_{r}\in R^{d^{y}},\;r=1,\ldots ,R$ be a vectorization of each frame of the raw movie. We assume that the movie has a low-rank nature and set the dimension of the hidden process *d*^*κ*^ to be a relatively small value compared to the dimension of each frame *d*^*y*^. Suppose that the external stimulus is also simultaneously measured and denoted as $x_{r}\in R^{d^{x}},\;r=1,\ldots ,R$. For notational simplicity, we denote these variables as $c={\left\{c_{r}\right\}}_{r=1}^{R}$, $y={\left\{y_{r}\right\}}_{r=1}^{R}$ and $x={\left\{x_{r}\right\}}_{r=1}^{R}$.

In previous studies [4, 6], the calcium fluorescence is expressed as convolution of the calcium ion decay with a spike train, which is considered as the 0–1 sequence. Conversely, in our method, we replace the 0–1 sequence with the marked spike sequence whose mark contains information of a fluorescent footprint emitted by each neuron. Let $\kappa_{r}\in R^{d^{\kappa }}\cup \left\{\emptyset \right\},\;r=2,\ldots ,R$ be this marked spike sequence; *κ*_*r*_ takes a vector of the fluorescent footprint added to the calcium fluorescence *c*_*r*_ if a neuron fires between the (*r* − 1)-th frame and *r*-th frame; otherwise, it takes ∅. We consider $\kappa ={\left\{\kappa_{r}\right\}}_{r=2}^{R}$. Given this ***κ***, we assume that the true calcium fluorescence ***c*** is distributed to a vector autoregressive model with jumps:

$$\begin{array}{cc} & c_{1}∼\text{Normal}\;(\mu_{\text{init}}^{c},{\left(\Sigma_{\text{init}}^{c}\right)}^{-1})\\ & \left\{ \begin{array}{cc} c_{r}=Fc_{r-1}+\kappa_{r}, & \text{(if}\;\kappa_{r}\neq \emptyset \text{)},\\ c_{r}=Fc_{r-1}+v_{r},\;v_{r}∼\text{Normal}\left(0,V\right), & \text{(if}\;\kappa_{r}=\emptyset \text{)}, \end{array}\right.\;r=2,\ldots ,R, \end{array}$$ (1)

where Normal(⋅) is a Gaussian distribution, $F\in R^{d^{\kappa }\times d^{\kappa }}$ is a transition matrix, $V\in R^{d^{\kappa }\times d^{\kappa }}$ is a transition precision matrix, $\mu_{\text{init}}^{c}$ is an initial state mean, and $\Sigma_{\text{init}}^{c}$ is an initial state covariance matrix. This assumption can be interpreted as follows. If any spike has not occurred, the calcium fluorescence *c*_*r*_ generally stays around zero, and small fluctuations induced by the noise *v*_*r*_ distribute to the Gaussian distribution whose precision matrix is *V*. If a spike has occurred between the (*r* − 1)-th frame and the *r*-th frame, then the fluorescent footprint of this spike *κ*_*r*_ is added to *c*_*r*_. The instantaneous increase of *c*_*r*_ due to this spike gradually decreases with the time constant decided by the transition matrix *F*.

Next, we consider that the movie ***y*** is observed as

$$\begin{array}{c} y_{r}=Gc_{r}+w_{r},\;w_{r}∼\text{Normal}\left(0,W\right),\;r=1,\ldots ,R, \end{array}$$ (2)

where $G\in R^{d^{y}\times d^{\kappa }}$ is an observation matrix, and $W\in R^{d^{y}\times d^{y}}$ is an observation precision matrix. The observation matrix *G* maps the calcium fluorescence *c*_*r*_ into the space of the images; *Gc*_*r*_ corresponds to the denoised frame of the movie, and *Gκ*_*r*_ corresponds to the vectorization of the fluorescent image of each spike. To reduce the computational cost in the estimation step, we restrict the observation precision matrix *W* as a diagonal matrix.

Therefore, the likelihood of ***κ***, given (***y***, ***c***), is

$$\begin{array}{cc} & p(y,c|\kappa )\\ & =N\left(c_{1}|\mu_{\text{init}}^{c},{\left(\Sigma_{\text{init}}^{c}\right)}^{-1}\right)\prod_{\kappa_{r}\neq \emptyset }\delta_{\kappa_{r}}\left(c_{r}-Fc_{r-1}\right)\prod_{\kappa_{r}=\emptyset }N\left(c_{r}-Fc_{r-1}|0,V\right)\prod_{r=1}^{R}N\left(y_{r}-Gc_{r}|0,W\right). \end{array}$$

#### Spike generation model

**Fig 10:**
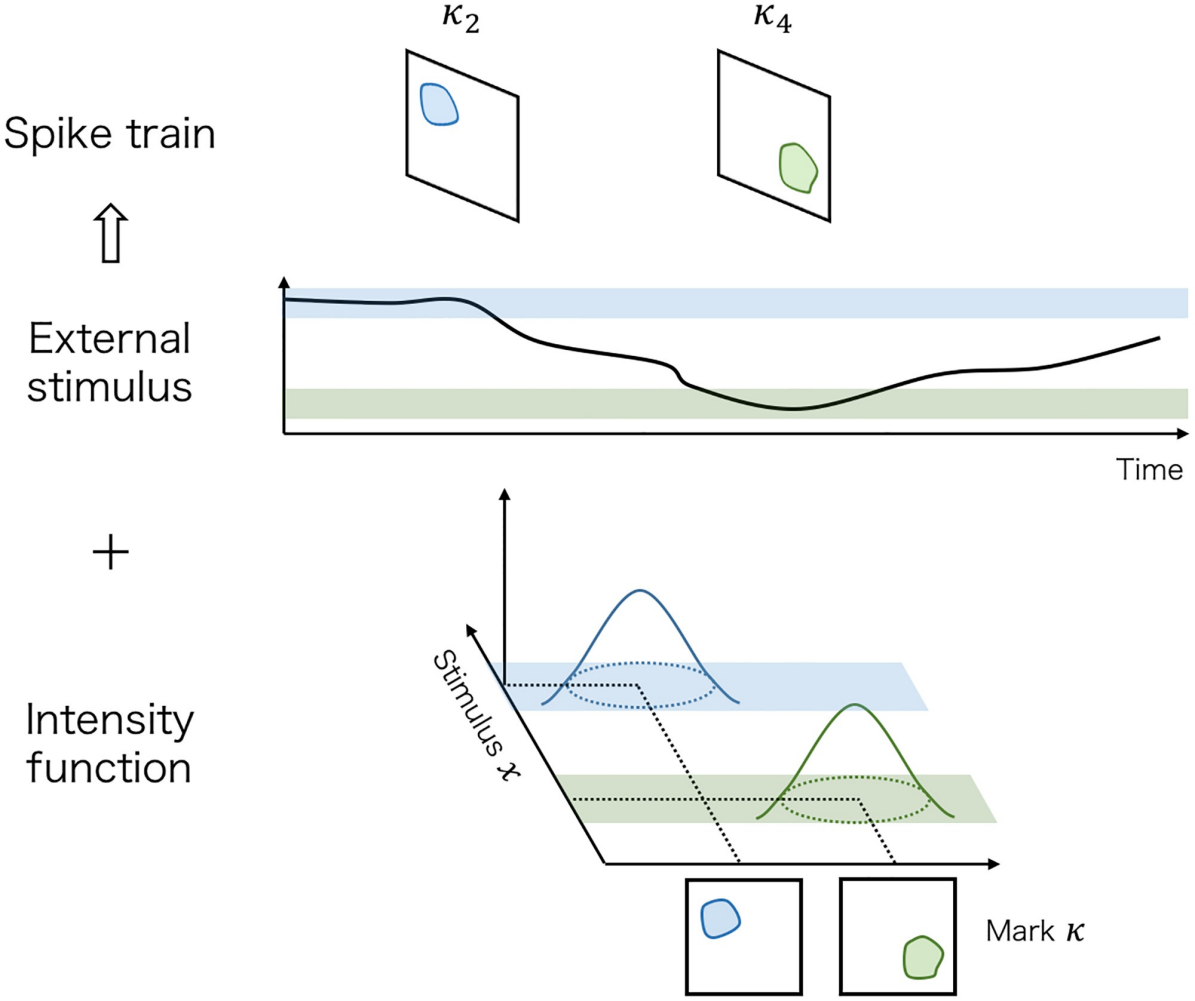
Spike generation model. Given the external stimulus ***x***, the marked spike sequences ***κ*** are generated from the marked point process whose intensity is λ(*κ* ∣ *x*, *ξ*). Each mark in these sequences indicates a fluorescent footprint of a spike emitted by each neuron, and this mark is expressed as a transformed version *Gκ*_*r*_ in this figure. Because each neuron in the movie has a characteristic fluorescent footprint and a characteristic receptive field for stimulus *x*, λ(*κ* ∣ *x*, *ξ*) can be expressed as the multimodal function with peaks corresponding to each neuron. The blue and green components indicate the mean florescent footprints and the receptive fields of two different neurons, respectively.

Next, we evaluate how the marked spike sequence is generated, given the external stimulus. In our model, we assume that the marked spike sequence ***κ*** is generated from marked point processes. Fig 10 illustrates this component.

A marked point process is a stochastic process that expresses a random occurrence of events (for details, see [21]). The probability structure of the point process is decided by a conditional intensity function

$$\begin{array}{cc} \lambda (t,\kappa |H_{t}) & ≔\lim_{\Delta t,\Delta \kappa \to 0}\frac{E[\begin{array}{c} \text{number}\;\text{of}\;\text{events}\;\text{whose}\;\text{mark}\;\text{within}\;\left(\kappa ,\kappa +\Delta \kappa \right]\\ \text{occurred}\;\text{at}\;\left(t,t+\Delta t\right] \end{array}\;|\;H_{t}]}{\Delta t\Delta \kappa }, \end{array}$$

where $\kappa \in R^{d^{\kappa }}$ is a mark attached to each event, and $H_{t}$ is the history of this process up to *t* that contains the information about external covariates. Using point process for modeling the series of events, we choose a family of conditional intensity function indexed by parameters and estimate these parameters given the observed data.

We consider that the marked spike sequences are generated from a spatio-temporal inhomogeneous Poisson process whose rate depends on the external stimuli *x*_*t*_. This means that the conditional intensity function is expressed as

$$\begin{array}{c} \lambda \left(t,\kappa |H_{t}\right)=\lambda \left(\kappa |x_{t},\xi \right) \end{array}$$

where λ(*κ* ∣ *x*, *ξ*) is a function defined on the product space $R^{d^{\kappa }}\times R^{d^{x}}$ and *ξ* is a parameter of the function. This λ(*κ* ∣ *x*, *ξ*) indicates how often spikes occur and what kinds of spikes occur depending on the external stimulus *x*; λ(*κ* ∣ *x*, *ξ*)Δ is the probability of observing a spike whose fluorescent footprint is *κ* in an interval with length Δ when the stimulus takes *x*.

What kind of parametric model is suitable for expressing this λ(*κ* ∣ *x*, *ξ*)? As explained in Introduction, when a neuron spikes, the calcium ion concentration inside this neuron rises sharply. Thus, each neuron emits a fluorescent footprint similar to its cell shape. Because we assume that the spiking rate is modulated by an external stimulus, each neuron also has a characteristic receptive field for the stimulus *x*. Therefore, the contribution of each neuron to λ(*κ* ∣ *x*, *ξ*) may become unimodal on the product space $R^{d^{\kappa }}\times R^{d^{x}}$. Hence, λ(*κ* ∣ *x*, *ξ*) can be expressed as a multimodal function of *κ* and *x* with peaks corresponding to each neuron.

Therefore, we model λ(*κ* ∣ *x*, *ξ*) as a mixture of kernel functions. Let *f*(*κ*, *x* ∣ *θ*) be a kernel function defined on $R^{d^{\kappa }\times d^{x}}$ indexed by *θ*, and let *ξ* be a finite measure defined on Θ. We assume that λ(*κ* ∣ *x*, *ξ*) is expressed as the integral *f*(*κ*, *x* ∣ *θ*) respect to *ξ* as

$$\begin{array}{cc} \lambda (\kappa |x,\xi ) & =\int_{\Theta }f\left(\kappa ,x|\theta \right)\;\xi \left(d\theta \right). \end{array}$$ (3)

Let *ξ* be a discrete measure defined on Θ, let ${\left\{u_{k}\right\}}_{k=1}^{\infty }$ be atoms of this *ξ*, and let ${\left\{\pi_{k}\right\}}_{k=1}^{\infty }$ be the weights attached to these atoms. Then, λ(*κ* ∣ *x*, *ξ*) in (3) is also expressed as

$$\begin{array}{c} \lambda \left(\kappa |x,\xi \right)=\sum_{k=1}^{\infty }\pi_{k}f\left(\kappa ,x|u_{k}\right). \end{array}$$

This expression can be interpreted as follows. An infinite number of neurons are influenced by the external stimulus. When the stimulus takes value *x*, the *k*-th neuron emits a spike whose mark is *κ* in a unit time interval with the probability *π*_*k*_*f*(*κ*, *x* ∣ *u*_*k*_). Note that the integral of *π*_*k*_*f*(*κ*, *x* ∣ *u*_*k*_) with respect to *κ* refers to the firing rate of the *k*-th neuron when the stimulus takes *x*, which is the tuning curve of this neuron.

In our model, we assume that *f*(*κ*, *x* ∣ *θ*) is expressed as the product of two kernels defined on $R^{d^{\kappa }}$ and $R^{d^{x}}$, respectively, as

$$\begin{array}{cc} f(\kappa ,x|\theta ) & =f^{\kappa }\left(\kappa |\theta^{\kappa }\right)\;f^{x}\left(x|\theta^{x}\right),\;\;\;\;\;\left(\theta^{\kappa },\theta^{x}\right)\in \Theta^{\kappa }\times \Theta^{x}. \end{array}$$

Here, *f*^*κ*^(*κ* ∣ *θ*^*κ*^) is the distribution of the fluorescent footprints emitted by a neuron, *f*^*x*^(*x* ∣ *θ*^*x*^) is the normalized tuning curve of the neuron, and *θ*^*κ*^ ∈ Θ^*κ*^ and *θ*^*x*^ ∈ Θ^*x*^ are the parameters of the kernels. This decomposition means that an external stimulus does not affect the shape of the fluorescent footprint of each spike. This assumption seems to be reasonable. To simplify the estimation procedure, we select a Gaussian kernel for *f*^*κ*^(*κ* ∣ *θ*^*κ*^):

$$\begin{array}{cc} f^{\kappa }\left(\kappa |\theta^{\kappa }\right) & =N\left(\kappa |\mu^{\kappa },\Lambda^{\kappa }\right),\;\;\;\;\;\theta^{\kappa }=\left(\mu^{\kappa },\Lambda^{\kappa }\right) \end{array}$$

where N(⋅ ∣ ⋅) is a Gaussian kernel defined on $R^{d^{\kappa }}$, *μ*^*κ*^ is a mean vector, and Λ^*κ*^ is a precision matrix about *κ*. For *f*^*x*^(*x* ∣ *θ*^*x*^), we select an appropriate kernel according to the type of stimulus we focus on. For example, if the stimulus takes a value on a Euclid space and the receptive field of the neuron has a unimodal shape, then a Gaussian kernel may be suitable. If the stimulus takes a value on the sphere, then the von Mises kernel is more appropriate for this *f*^*x*^(*x* ∣ *θ*^*x*^). For notational simplicity, we use these general notations for *f*^*κ*^ and *f*^*x*^ in the remainder of this paper.

The likelihood of *ξ*, given ***κ***, is expressed under the notion of the point process. In our model, we use a discretized expression for the point process. Consider that the sampling interval Δ is sufficiently small and assume that each bin ((*r* − 1)Δ, *r*Δ], *r* = 1, …, *R* contains at most one spike. Under this assumption, the probability of observing a spike whose footprint is *κ*_*r*_ in ((*r* − 1)Δ, *r*Δ] is λ(*κ*_*r*_ ∣ *x*_*r*_, *ξ*)Δ and that of not observing any spike in ((*r* − 1)Δ, *r*Δ] is 1 − ∫λ(*κ* ∣ *x*_*r*_, *ξ*)d*κ* Δ. Multiplying these probabilities for the all bins and approximating the term 1 − ∫λ(*κ* ∣ *x*_*r*_, *ξ*)d*κ* Δ as

$$\begin{array}{c} 1-\int \lambda \left(\kappa |x_{r},\xi \right)d\kappa \;\Delta \approx \exp(-\int_{(r-1)\Delta }^{r\Delta }\int_{R^{d^{\kappa }}}\lambda \left(\kappa |x_{t},\xi \right)\;d\kappa \;dt), \end{array}$$

we obtain the likelihood of *ξ*, given ***κ***, as

$$\begin{array}{cc} p(\kappa |\xi ) & =\exp(-\int_{0}^{T}\int_{R^{d^{\kappa }}}\lambda \left(\kappa |x_{t},\xi \right)\;d\kappa \;dt)\prod_{\kappa_{r}\neq \emptyset }(\lambda (\kappa_{r}|x_{r},\xi )\;\Delta )\\ & =\exp(-T\int_{R^{d^{x}}}\int_{R^{d^{\kappa }}}\int_{\Theta^{x}}\int_{\Theta^{\kappa }}f^{\kappa }\left(\kappa |\theta^{\kappa }\right)\;f^{x}\left(x|\theta^{x}\right)\;\xi \left(d\theta^{\kappa },d\theta^{x}\right)\;d\kappa \;\eta \left(dx\right))\\ & \;\cdot \prod_{\kappa_{r}\neq \emptyset }(\int_{\Theta^{x}}\int_{\Theta^{\kappa }}f^{\kappa }\left(\kappa_{r}|\theta^{\kappa }\right)\;f^{x}\left(x_{r}|\theta^{x}\right)\;\xi \left(d\theta^{\kappa },d\theta^{x}\right)\;\Delta ). \end{array}$$

Here, *η* is an empirical measure of {*x*_*t*_}_*t*∈(0,*T*]_ approximated by

$$\begin{array}{c} \eta \approx \frac{1}{R}\sum_{r=1}^{R}\delta_{x_{r}}. \end{array}$$

For the sake of notational simplicity, we omit the conditioning by ***x*** in the following equation.

#### Prior

To estimate *ξ* and other hyperparameters from the data, we take a Bayesian approach. In other words, we set a prior for *ξ* and calculate a posterior of *ξ* given the observed data. Specifically, we adopt a mixture of weighted gamma processes as a prior for *ξ*.

A weighted gamma process is a stochastic process defined on the space of finite discrete measures. A random measure *ξ*_*α*_ is said to be distributed according to a gamma process with parameter *α* if

$$\begin{array}{c} \xi_{\alpha }\left(A\right)∼\text{Gamma}\left(\alpha \left(A\right)\right),\;A\subset \Theta \end{array}$$

where *α* is a finite measure on Θ, and Gamma(*α*(*A*)) is a gamma distribution with mean and variance *α*(*A*). A random measure *ξ*_*α*,*β*_ is said to be distributed according to a weighted gamma process with parameters *α* and *β* if

$$\begin{array}{c} \xi_{\alpha ,\beta }\left(A\right)=\int_{A}\beta \left(\theta \right)\;\xi_{\alpha }\left(d\theta \right),\;A\subset \Theta \end{array}$$

where *β* is a nonnegative function defined on Θ. These processes can be considered as random variables defined on the space M(Θ) that is composed of all finite measures on Θ. The probability measure of *ξ*_*α*,*β*_ defined on M(Θ) is denoted as G(dξ∣α,β). It is known that this process can be used as a prior for intensity estimation [22, 23, 24].

Although a weighted gamma process is also a conjugate prior for *ξ* in our model, it is difficult to handle the posterior expressed as this process because the weighted gamma process generates a random discrete measure whose support consists of an infinite number of atoms, and we cannot simulate samples from this process on the computer. [24] addressed this problem by using a mixture of gamma processes instead of the weighted gamma process.

In our model, we modify the definition of the mixture of gamma processes in [24]. We define $G_{K}\left(\cdot \right)$ as

$$\begin{array}{cc} G_{K}(d\xi |\alpha_{0},\beta_{0},H,u^{x}) & =\int G(d\xi \;|\;\frac{\alpha_{0}}{K}\sum_{k=1}^{K}\delta_{\left(u_{k}^{\kappa },u_{k}^{x}\right)},\beta_{0})\;\prod_{k=1}^{K}H^{\kappa }\left(du_{k}^{\kappa }\right), \end{array}$$

where *α*_0_ > 0 and *β*_0_ > 0 are scalar parameters, $u^{\kappa }={\left\{u_{k}^{\kappa }\right\}}_{k=1}^{K},\;u^{x}={\left\{u_{k}^{x}\right\}}_{k=1}^{K}$ are kernel parameters, and *H*^*κ*^ is a prior for $u^{\kappa }$ defined by

$$\begin{array}{cc} \frac{H^{\kappa }\left(du^{\kappa }\right)}{du^{\kappa }} & =\text{NW}(\mu^{\kappa },\Lambda^{\kappa }|m^{\kappa },\gamma^{\kappa },\nu^{\kappa },S^{\kappa }). \end{array}$$

Here, NW(⋅∣⋅) is a normal-Wishart distribution, with location vector *m*^*κ*^ and scale matrix *S*^*κ*^. The parameters *γ*^*κ*^ > 0 and *ν*^*κ*^ > *d*^*κ*^ − 1 are scalars. We formulate $u^{x}$ as hyperparameters. Because the dimension of $u_{k}^{x}$ is relatively small in our problem, this formulation works reasonably well. In the estimation step, we calculate the posterior of $u^{\kappa }$ and also estimate $u^{x}$ in the empirical Bayes approach.

**Fig 11:**
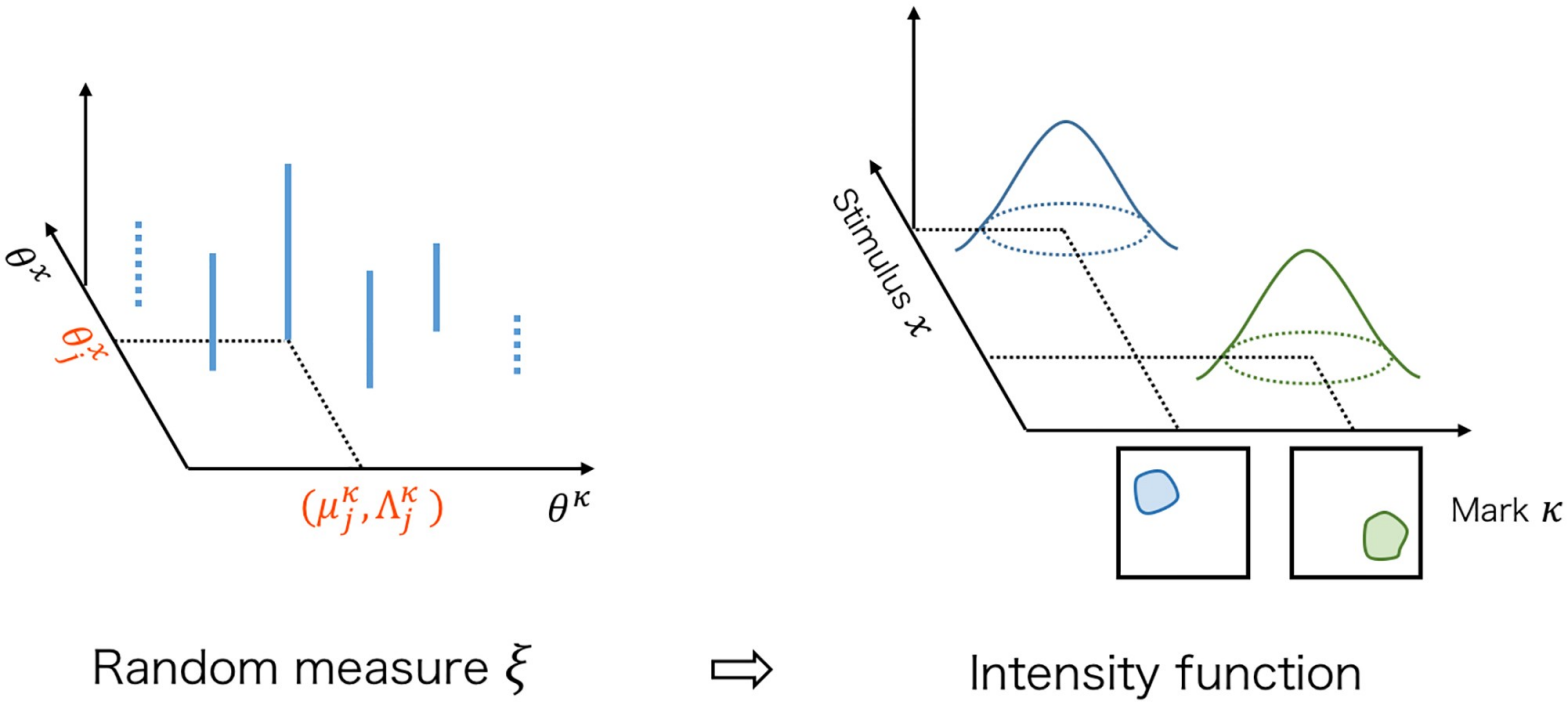
Prior. Intensity function λ(*κ* ∣ *x*, *ξ*) is expressed as the sum of kernels, and it is indexed by the discrete measure *ξ* defined on Θ^*κ*^ × Θ^*x*^. This discrete measure *ξ* consists of atoms $\left\{\left(\mu_{k}^{\kappa },\Lambda_{k}^{\kappa },\theta_{k}^{x}\right)\right\}$ and each $\mu_{k}^{\kappa },\Lambda_{k}^{\kappa },\theta_{k}^{x}$ corresponds to the mean fluorescent image, the precision matrix of the fluorescent images, and the tuning curve parameter of each neuron. A weight attached to each atom expresses the average firing rate of this neuron. We set the mixture of gamma processes $G_{K}\left(d\xi |\alpha_{0},\beta_{0},H,u^{x}\right)$ as a prior for this *ξ*.

Fig 11 shows how *ξ* and the intensity function are related to each other.

### Another expression of the generative model

To construct an efficient estimation procedure, we provide another expression of our generative model.

First, we introduce a new latent variable. Because we set the mixture of weighted gamma processes as a prior for *ξ*, each atom of *ξ* corresponds to one of $(u_{k}^{\kappa },u_{k}^{x}),\;k=1,\ldots ,K$. Here, we introduce a new latent variable ***z*** = {*z*_*rk*_}_*r* = 2, …, *R*; *k* = 1, …, *K*_ indicating which of the parameters $(u_{k}^{\kappa },u_{k}^{x}),\;k=1,\ldots ,K$ generates *κ*_*r*_. We define ***z*** as

$$\begin{array}{cc} & z_{rk}=\{\begin{array}{cc} 1, & \text{if}\;\text{the}\;\text{kernel}\;\text{with}\;\text{parameter}\;\left(u_{k}^{\kappa },u_{k}^{x}\right)\;\text{generates}\;\kappa_{r},\\ 0, & \text{otherwise}, \end{array}\\ & r=2,\ldots ,R;\;k=1,\ldots ,K. \end{array}$$

Note that if *κ*_*r*_ = ∅ at *r*, all of *z*_*rk*_, *k* = 1, …, *K* are 0.

The likelihood function of *ξ* given (***κ***, ***z***) is

$$\begin{array}{cc} p(\kappa ,z|\xi ) & =\exp(-T\int_{R^{d^{x}}}\int_{R^{d^{\kappa }}}\int_{\Theta^{x}}\int_{\Theta^{\kappa }}f^{\kappa }\left(\kappa |\theta^{\kappa }\right)\;f^{x}\left(x|\theta^{x}\right)\;\xi \left(d\theta^{\kappa },d\theta^{x}\right)\;d\kappa \;\eta \left(dx\right))\\ & \;\cdot \prod_{\kappa_{r}\neq \emptyset }\prod_{k=1}^{K}{\left(f^{\kappa }\left(\kappa_{r}|u_{k}^{\kappa }\right)\;f^{x}\left(x_{r}|u_{k}^{x}\right)\;\xi \left(du_{k}^{\kappa },du_{k}^{x}\right)\;\Delta \right)}^{z_{rk}}. \end{array}$$

The complete likelihood of *ξ* is

$$\begin{array}{cc} & p(y,c,\kappa ,z|\xi )\\ & =p(y,c|\kappa )\;p(\kappa ,z|\xi )\\ & =N\left(c_{1}|\mu_{\text{init}}^{c},{\left(\Sigma_{\text{init}}^{c}\right)}^{-1}\right)\prod_{\kappa_{r}\neq \emptyset }\delta_{\kappa_{r}}\left(c_{r}-Fc_{r-1}\right)\prod_{\kappa_{r}=\emptyset }N\left(c_{r}-Fc_{r-1}|0,V\right)\prod_{r=1}^{R}N\left(y_{r}-Gc_{r}|0,W\right)\\ & \;\cdot \exp(-T\int_{R^{d^{x}}}\int_{R^{d^{\kappa }}}\int_{\Theta^{x}}\int_{\Theta^{\kappa }}f^{\kappa }\left(\kappa |\theta^{\kappa }\right)\;f^{x}\left(x|\theta^{x}\right)\;\xi \left(d\theta^{\kappa },d\theta^{x}\right)\;d\kappa \;\eta \left(dx\right))\\ & \;\cdot \prod_{\kappa_{r}\neq \emptyset }\prod_{k=1}^{K}{\left(f^{\kappa }\left(\kappa_{r}|u_{k}^{\kappa }\right)\;f^{x}\left(x_{r}|u_{k}^{x}\right)\;\xi \left(du_{k}^{\kappa },du_{k}^{x}\right)\;\Delta \right)}^{z_{rk}}. \end{array}$$

Since the equality *κ*_*r*_ = *c*_*r*_ − *Fc*_*r*−1_ holds when *κ*_*r*_ ≠ ∅, this likelihood depends on *κ*_*r*_ only through *c*_*r*_ and *c*_*r*−1_. Therefore, we can remove ***κ*** from this equation. The likelihood of *ξ* can be expressed as

$$\begin{array}{cc} & p(y,c,z|\xi )\\ & =N\left(c_{1}|\mu_{\text{init}}^{c},{\left(\Sigma_{\text{init}}^{c}\right)}^{-1}\right)\prod_{r=2}^{R}N{\left(c_{r}-Fc_{r-1}|0,V\right)}^{\left(1-\sum_{k=1}^{K}z_{rk}\right)}\prod_{r=1}^{R}N\left(y_{r}-Gc_{r}|0,W\right)\\ & \;\cdot \exp(-T\int_{R^{d^{x}}}\int_{R^{d^{\kappa }}}\int_{\Theta^{x}}\int_{\Theta^{\kappa }}f^{\kappa }\left(\kappa |\theta^{\kappa }\right)\;f^{x}\left(x|\theta^{x}\right)\;\xi \left(d\theta^{\kappa },d\theta^{x}\right)\;d\kappa \;\eta \left(dx\right))\\ & \;\cdot \prod_{r=2}^{R}\prod_{k=1}^{K}{\left(f^{\kappa }\left(c_{r}-Fc_{r-1}|u_{k}^{\kappa }\right)\;f^{x}\left(x_{r}|u_{k}^{x}\right)\;\xi \left(du_{k}^{\kappa },du_{k}^{x}\right)\;\Delta \right)}^{z_{rk}}. \end{array}$$

Under this likelihood, the posterior of the hidden variables is characterized by the following proposition.

**Proposition 1**. *Under our generative model and the prior*

$$\begin{array}{c} G_{K}\left(d\xi |\alpha_{0},\beta_{0},H^{\kappa },u^{x}\right), \end{array}$$

*the posterior of (**c**, **z**, ξ), given* $u^{x}$ *and **y**, is*

$$\begin{array}{c} p\left(dc,dz,d\xi |u^{x},y\right)=\int G(d\xi \;|\;\sum_{k=1}^{K}(\frac{\alpha_{0}}{K}+\sum_{r=2}^{R}z_{rk})\delta_{\left(u_{k}^{\kappa },u_{k}^{x}\right)},\beta^{*})p\left(dc,dz,du^{\kappa }|u^{x},y\right). \end{array}$$

*Here*, $p(dc,dz,du^{\kappa }|u^{x},y)$ *is the posterior of* $\left(c,z,u^{\kappa }\right)$, *given* $u^{x}$ *and **y**, that is proportional to*

$$\begin{array}{cc} & p(dc,dz,du^{\kappa }|u^{x},y)\\ & \propto N\left(c_{1}|\mu_{\text{init}}^{c},{\left(\Sigma_{\text{init}}^{c}\right)}^{-1}\right)\prod_{r=2}^{R}N{\left(c_{r}-Fc_{r-1}|0,V\right)}^{\left(1-\sum_{k=1}^{K}z_{rk}\right)}\;\prod_{r=1}^{R}N\left(y_{r}-Gc_{r}|0,W\right)\\ & \;\cdot \prod_{r=2}^{R}\prod_{k=1}^{K}{\left(\beta^{*}\left(u_{k}^{x}\right)\;f^{\kappa }\left(c_{r}-Fc_{r-1}|u_{k}^{\kappa }\right)\;f^{x}\left(x_{r}|u_{k}^{x}\right)\;\Delta \right)}^{z_{rk}}\\ & \;\cdot (\int (\prod_{r=2}^{R}\prod_{k=1}^{K}P{\left(du_{k}^{\kappa },du_{k}^{x}\right)}^{z_{rk}})DP(dP\;|\;\frac{\alpha_{0}}{K}\sum_{k=1}^{K}\delta_{\left(u_{k}^{\kappa },u_{k}^{x}\right)}))\;dc\;dz\;\prod_{k=1}^{K}H^{\kappa }\left(du_{k}^{\kappa }\right), \end{array}$$

*where*

$$\begin{array}{cc} \beta^{*}\left(\theta^{x}\right) & =\frac{\beta_{0}}{1+\beta_{0}T\int_{R^{d^{x}}}f^{x}\left(x|\theta^{x}\right)\eta \left(dx\right)} \end{array}$$

*and* $DP(dP|\frac{\alpha_{0}}{K}\sum_{k=1}\delta_{\left(u_{k}^{\kappa },u_{k}^{x}\right)})$ *is a Dirichlet process with base measure* $\frac{\alpha_{0}}{K}\sum_{k=1}^{K}\delta_{\left(u_{k}^{\kappa },u_{k}^{x}\right)}$. *A random measure P distributed according to* $DP(dP|\frac{\alpha_{0}}{K}\sum_{k=1}\delta_{\left(u_{k}^{\kappa },u_{k}^{x}\right)})$ *can be expressed as*

$$\begin{array}{cc} P & \overset{d}{=}\sum_{k=1}^{K}\pi_{k}\delta_{\left(u_{k}^{\kappa },u_{k}^{x}\right)},\\ \pi =(\pi_{1},\ldots ,\pi_{K}) & ∼\text{Dir}(\frac{\alpha_{0}}{K},\ldots ,\frac{\alpha_{0}}{K}). \end{array}$$

*This distribution* $p(dc,dz,du^{\kappa }|u^{x},y)$ *can be expressed as the marginal distribution by introducing an additional hidden variable **π*** = (*π*_1_, …, *π*_*K*_) *as*

$$\begin{array}{c} p\left(dc,dz,du^{\kappa }|u^{x},y\right)=\int_{S^{K-1}}p\left(dc,dz,d\pi ,du^{\kappa }|u^{x},y\right). \end{array}$$

*Here*, $p(dc,dz,d\pi ,du^{\kappa }|u^{x},y)$ *is the posterior of* $\left(c,z,\pi ,u^{\kappa }\right)$, *given* $u^{x}$ *and **y**, that is proportional to*

$$\begin{array}{cc} & p(dc,dz,d\pi ,du^{\kappa }|u^{x},y)\\ & \propto N\left(c_{1}|\mu_{\text{init}}^{c},{\left(\Sigma_{\text{init}}^{c}\right)}^{-1}\right)\prod_{r=2}^{R}N{\left(c_{r}-Fc_{r-1}|0,V\right)}^{\left(1-\sum_{k=1}^{K}z_{rk}\right)}\;\prod_{r=1}^{R}N(y_{r}-Gc_{r}|0,W)\\ & \;\cdot \prod_{r=2}^{R}\prod_{k=1}^{K}{\left(\beta^{*}(u_{k}^{x})\;f^{\kappa }(c_{r}-Fc_{r-1}|u_{k}^{\kappa })\;f^{x}(x_{r}|u_{k}^{x})\;\Delta \right)}^{z_{rk}}\\ & \;\cdot \prod_{r=2}^{R}\prod_{k=1}^{K}\pi_{k}^{z_{rk}}\;\text{Dir}(\pi \;|\;\frac{\alpha_{0}}{K},\ldots ,\frac{\alpha_{0}}{K})\;dc\;dz\;d\pi \;\prod_{k=1}^{K}H^{\kappa }\left(du_{k}^{\kappa }\right). \end{array}$$

The proof of this proposition is given in S1 Appendix. This proposition enables us to calculate the marginal posterior of $\left(c,z,u^{\kappa }\right)$ without considering *ξ* by using the efficient estimation procedure described in the next section.

### Variational inference

To obtain the approximate posterior, we use a variational inference approach [25, 26]. The log marginal likelihood of ***y*** can be decomposed as

$$\begin{array}{cc} \log p(y) & =L(q)+\text{KL}(q\;||\;p),\\ L(q) & ≔E_{q(c,z,\pi ,u^{\kappa })}[\log\frac{p(y,c,z,\pi ,u^{\kappa }|u^{x})}{q(c,z,\pi ,u^{\kappa })}],\\ \text{KL}(q\;||\;p) & ≔E_{q(c,z,\pi ,u^{\kappa })}[\log\frac{q(c,z,\pi ,u^{\kappa })}{p(c,z,\pi ,u^{\kappa }|u^{x},y)}]. \end{array}$$

Here, *q* is an arbitrary distribution of $\left(c,z,\pi ,u^{\kappa }\right)$, and KL(*q*||*p*) is the Kullback–Leibler divergence between *q* and the true posterior $p(c,z,\pi ,u^{\kappa }|u^{x},y)$. Because the left-hand side of this equation is independent of *q*, maximization of L(q) with respect to *q* corresponds to minimization of the Kullback–Leibler divergence between *q* and the true posterior. If *q* is the true posterior, then L(q) takes the maximum value. This L(q) is called the evidence lower bound.

However, it is difficult to calculate L(q) for an arbitrary *q*. Therefore, we specify the distribution family Q that makes it is easy to calculate L(q), and find the distribution with the largest L(q) among this family. In other words, we solve the constrained functional optimization problem

$$\begin{array}{c} \underset{q\in Q}{\text{arg}\;\text{max}}L\left(q\right) \end{array}$$

and consider the solution of this problem as the approximation of the true posterior.

In this paper, we introduce an independence constraint

$$\begin{array}{cc} q(c,z,\pi ,u^{\kappa }) & =q\left(c\right)\;q\left(z\right)\;q\left(\pi \right)\;q\left(u_{k}^{\kappa }\right). \end{array}$$

Under this constraint, we maximize L(q) using coordinate ascent maximization. In the estimation step, we update the posterior of each parameter to the distribution that maximizes L(q), while keeping the distributions of the other variables as fixed. Under our model, the following distribution families satisfy the stationary condition of the functional optimization problem:

$$\begin{array}{cc} q(c) & =N(c|{\left\{\mu_{r}^{c}\right\}}_{r=1}^{R},{\left\{\Sigma_{r}^{c}\right\}}_{r=1}^{R},{\left\{\Sigma_{r-1,r}^{c}\right\}}_{r=2}^{R}),\\ q(z) & =\prod_{r=2}^{R}{\left(1-\sum_{k=1}^{K}\zeta_{rk}\right)}^{\left(1-\sum_{k=1}^{K}z_{rk}\right)}\prod_{k=1}^{K}\zeta_{rk}^{z_{rk}},\\ q(\pi ) & =\text{Dir}(\pi |\alpha_{1},\ldots ,\alpha_{K}),\\ q(u^{\kappa }) & =\prod_{k=1}^{K}q\left(u_{k}^{\kappa }\right)=\prod_{k=1}^{K}\text{NW}\left(\mu_{k}^{\kappa },\Lambda_{k}^{\kappa }|m_{k}^{\kappa },\gamma_{k}^{\kappa },\nu_{k}^{\kappa },S_{k}^{\kappa }\right). \end{array}$$

The details of these distributions are shown in S1 Appendix. Therefore, instead of numerically updating the distribution, we can maximize L(q) with respect to the parameters of these distribution families.

At each iteration, we update the distribution of each parameter one by one and also maximize L(q) with respect to the hyperparameters. We repeat this operation several times and obtain the variational posterior $\hat{q}$. By calculating the variational expectation of the hidden variables using this $\hat{q}$, we obtain the deconvolution result.

## Supporting information

S1 Appendix Technical appendix. The supplementary material includes the technical details of our model. We provide a proof for Proposition 2, the updated formulas in the estimation step, implication of the obtained posterior under our model, and some heuristic techniques for obtaining a good posterior approximation.
(PDF)
