# Supplementary material for: Deconvolution of calcium imaging data using marked point processes
Source: PLoS Comput Biol. 2020 Mar 12;16(3):e1007650. doi: 10.1371/journal.pcbi.1007650 (PMC7093033; doi:10.1371/journal.pcbi.1007650)
Supplement: S1 Appendix — The supplementary material includes the technical details of our model. We provide a proof for Proposition 2, the updated formulas in the estimation step, implication of the obtained posterior under our model, and some heuristic techniques for obtaining a good posterior approximation. (PDF) [file pcbi.1007650.s001.pdf]

S1 Appendix. Technical appendix

1 Distribution

In this section, we give the definitions of probability density function used in this paper.

- Univariate Gaussian distribution

$$\mathrm{N}(x \mid \mu, \tau) = \sqrt{\frac{\tau}{2\pi}} \exp\left(-\frac{\tau}{2}(x-\mu)^2\right), \quad x \in \mathbb{R}.$$

- Multivariate Gaussian distribution

$$\mathrm{N}(\kappa \mid \mu, \Lambda) = \frac{|\Lambda|^{\frac{1}{2}}}{(2\pi)^{\frac{d}{2}}} \exp\left(-\frac{1}{2}(\kappa-\mu)^{\mathrm{T}}\Lambda(\kappa-\mu)\right), \quad \kappa \in \mathbb{R}^d.$$

- Wishart distribution

$$\mathrm{W}(\Lambda \mid \nu, S) = \frac{|\Lambda|^{\frac{\nu-d-1}{2}}}{|S|^{\frac{\nu}{2}} 2^{\frac{\nu d}{2}} \pi^{\frac{d(d-1)}{4}} \prod_{i=1}^{d} \Gamma\left(\frac{\nu+1-i}{2}\right)} \exp\left(-\frac{1}{2}\mathrm{tr}\left(S^{-1}\Lambda\right)\right), \quad \Lambda \in \mathbb{R}^{d\times d}.$$

- Normal-Wishart distribution

$$\mathrm{NW}\left(\mu, \Lambda \,|\, m, \gamma, \nu, S\right) = \mathrm{N}\left(\mu \,|\, m, \gamma\Lambda\right)\, \mathrm{W}\left(\Lambda \,|\, \nu, S\right), \quad \mu \in \mathbb{R}^d,\ \Lambda \in \mathbb{R}^{d\times d}.$$

- Multivariate Student's t distribution

$$\mathrm{St}(\kappa \mid m, S, \nu) = \frac{\Gamma\left(\frac{\nu+d}{2}\right)|S|^{\frac{1}{2}}}{\Gamma\left(\frac{\nu}{2}\right)\nu^{\frac{d}{2}}\pi^{\frac{d}{2}}} \left(1 + \frac{1}{\nu}(\kappa-m)^{\mathrm{T}}S(\kappa-m)\right)^{-\frac{\nu+d}{2}}, \quad \kappa \in \mathbb{R}^d.$$

- Gamma distribution

$$\mathrm{Gam}(x \mid \alpha) = \frac{1}{\Gamma(\alpha)} x^{\alpha-1} \exp\left(-x\right), \quad x \in \mathbb{R}_+.$$

- Dirichlet distribution

$$\mathrm{Dir}(\boldsymbol{\pi} \mid \alpha_1, \ldots, \alpha_K) = \frac{\Gamma\left(\sum_{k=1}^K \alpha_k\right)}{\prod_{k=1}^K \Gamma(\alpha_k)} \prod_{k=1}^K \pi_k^{\alpha_k - 1}, \quad \boldsymbol{\pi} \in S^{K-1}.$$

- von Mises distribution

$$\mathrm{VM}(x \mid \mu, \tau) = \frac{1}{2\pi I_0(\tau)} \exp\left(\tau \cos(x-\mu)\right), \quad -\pi \leq x \leq \pi,$$

where I_0 is the modified Bessel function of order 0.

2 Proof of the proposition

In this section, we give the proof for Proposition 1 in section 4.

Proof of Proposition 1. Instead of calculating the posterior directly, we calculate the posterior mean of an arbitrary integrable function $g(\boldsymbol{c}, \boldsymbol{z}, \xi)$ with respect to the posterior

$$\int g(\boldsymbol{c}, \boldsymbol{z}, \xi)\, p(\mathrm{d}\boldsymbol{c}, \mathrm{d}\boldsymbol{z}, \mathrm{d}\xi \mid \mathbb{u}^x, \boldsymbol{y}) \tag{1}$$

and show that this value corresponds to

$$\frac{\int g(\boldsymbol{c}, \boldsymbol{z}, \xi)\, p(\boldsymbol{y}, \boldsymbol{c}, \boldsymbol{z} \mid \xi) \mathrm{d}\boldsymbol{c}\, \mathrm{d}\boldsymbol{z}\, \mathcal{G}_K(\mathrm{d}\xi \mid \alpha_0, \beta_0, H^\kappa, \mathbb{u}^x)}{\int p(\boldsymbol{y}, \boldsymbol{c}, \boldsymbol{z} \mid \xi)\, \mathrm{d}\boldsymbol{c}\, \mathrm{d}\boldsymbol{z}\, \mathcal{G}_K(\mathrm{d}\xi \mid \alpha_0, \beta_0, H^\kappa, \mathbb{u}^x)}. \tag{2}$$

First, we calculate the numerator of (2). From the definition of the mixture of gamma

processes and the likelihood of ξ,

$$\int g(\boldsymbol{c}, \boldsymbol{z}, \xi)\, p(\boldsymbol{y}, \boldsymbol{c}, \boldsymbol{z} \mid \xi)\, \mathrm{d}\boldsymbol{c}\, \mathrm{d}\boldsymbol{z}\, \mathcal{G}_K(\mathrm{d}\xi \mid \alpha_0, \beta_0, H^\kappa, \mathbb{u}^x)$$

$$= \int g(\boldsymbol{c}, \boldsymbol{z}, \xi)\, \exp\left(-T \int_{\mathbb{R}^{d^x}} \int_{\mathbb{R}^{d^\kappa}} \int_{\Theta^x} \int_{\Theta^\kappa} f^\kappa(\kappa \mid \theta^\kappa)\, f^x(x \mid \theta^x)\, \xi(\mathrm{d}\theta^\kappa, \mathrm{d}\theta^x)\, \mathrm{d}\kappa\, \eta(\mathrm{d}x)\right)$$

$$\cdot \mathrm{N}(c_1 \mid \mu^c_{\text{init}}, (\Sigma^c_{\text{init}})^{-1}) \prod_{r=2}^{R} \mathrm{N}\left(c_r - F c_{r-1} \mid 0, V\right)^{\left(1 - \sum_{k=1}^{K} z_{rk}\right)} \prod_{r=1}^{R} \mathrm{N}(y_r - G c_r \mid 0, W)$$

$$\cdot \prod_{r=2}^{R} \prod_{k=1}^{K} \left(f^\kappa(c_r - F c_{r-1} \mid u^\kappa_k)\, f^x(x_r \mid u^x_k)\, \xi(\mathrm{d}u^\kappa_k, \mathrm{d}u^x_k)\, \Delta\right)^{z_{rk}}$$

$$\mathrm{d}\boldsymbol{c}\, \mathrm{d}\boldsymbol{z}\, \mathcal{G}\left(\mathrm{d}\xi \,\middle|\, \frac{\alpha_0}{K} \sum_{k=1}^{K} \delta_{(u^\kappa_k, u^x_k)}, \beta_0\right) \prod_{k=1}^{K} H^\kappa(\mathrm{d}u^\kappa_k).$$

Using the lemma 1 in [1], we can move the exponential term outside of the integral as

$$\exp\left(-\sum_{k=1}^{K} \frac{\alpha_0}{K} \log\left(1 + \beta_0 T \int_{\mathbb{R}^{d^x}} f^x(x \mid u^x_k)\, \eta(\mathrm{d}x)\right)\right)$$

$$\int\int g(\boldsymbol{c}, \boldsymbol{z}, \xi)\, \mathrm{N}(c_1 \mid \mu^c_{\text{init}}, (\Sigma^c_{\text{init}})^{-1}) \prod_{r=2}^{R} \mathrm{N}\left(c_r - F c_{r-1} \mid 0, V\right)^{\left(1 - \sum_{k=1}^{K} z_{rk}\right)} \prod_{r=1}^{R} \mathrm{N}(y_r - G c_r \mid 0, W)$$

$$\cdot \prod_{r=2}^{R} \prod_{k=1}^{K} \left(f^\kappa(c_r - F c_{r-1} \mid u^\kappa_k)\, f^x(x_r \mid u^x_k)\, \xi(\mathrm{d}u^\kappa_k, \mathrm{d}u^x_k)\, \Delta\right)^{z_{rk}}$$

$$\mathrm{d}\boldsymbol{c}\, \mathrm{d}\boldsymbol{z}\, \mathcal{G}\left(\mathrm{d}\xi \,\middle|\, \frac{\alpha_0}{K} \sum_{k=1}^{K} \delta_{(u^\kappa_k, u^x_k)}, \beta^*\right) \prod_{k=1}^{K} H^\kappa(\mathrm{d}u^\kappa_k).$$

Here, the definition of β^* is shown in the main paper.

Next, we focus on the integration in the above equation. The integration respect to $\boldsymbol{z}$ is a summation respect to all combination of $\boldsymbol{z}$. Since $\boldsymbol{z}$ takes only a finite number of combinations,

this summation can be moved outside of the integral with respect to the gamma process as

$$\int g(\boldsymbol{c}, \boldsymbol{z}, \xi)\, \mathrm{N}(c_1 \mid \mu^c_{\mathrm{init}}, (\Sigma^c_{\mathrm{init}})^{-1}) \prod_{r=2}^{R} \mathrm{N}\left(c_r - Fc_{r-1} \mid 0, V\right)^{\left(1-\sum_{k=1}^{K} z_{rk}\right)} \prod_{r=1}^{R} \mathrm{N}(y_r - Gc_r \mid 0, W)$$
$$\cdot \prod_{r=2}^{R}\prod_{k=1}^{K} (f^{\kappa}(c_r - Fc_{r-1} \mid u^{\kappa}_k)\, f^{x}(x_r \mid u^{x}_k)\, \xi(\mathrm{d}u^{\kappa}_k, \mathrm{d}u^{x}_k)\, \Delta)^{z_{rk}}$$
$$\mathrm{d}\boldsymbol{c}\, \mathrm{d}\boldsymbol{z}\, \mathcal{G}\left(\mathrm{d}\xi \,\middle|\, \frac{\alpha_0}{K}\sum_{k=1}^{K} \delta_{(u^{\kappa}_k, u^{x}_k)}, \beta^*\right) \prod_{k=1}^{K} H^{\kappa}(\mathrm{d}u^{\kappa}_k)$$
$$= \int \mathrm{N}(c_1 \mid \mu^c_{\mathrm{init}}, (\Sigma^c_{\mathrm{init}})^{-1}) \prod_{r=1}^{R} \mathrm{N}(y_r - Gc_r \mid 0, W)$$
$$\cdot \sum_{\boldsymbol{z}} \left(B(\boldsymbol{c}, \boldsymbol{z}) \prod_{r=2}^{R} \mathrm{N}(c_r - Fc_{r-1} \mid 0, V)^{\left(1-\sum_{k=1}^{K} z_{rk}\right)} \right) \mathrm{d}\boldsymbol{c},$$

where

$$B(\boldsymbol{c}, \boldsymbol{z}) = \int_{\mathcal{M}(\Theta)} g(\boldsymbol{c}, \boldsymbol{z}, \xi) \prod_{r=2}^{R}\prod_{k=1}^{K} (f^{\kappa}(c_r - Fc_{r-1} \mid u^{\kappa}_k)\, f^{x}(x_r \mid u^{x}_k)\, \xi(\mathrm{d}u^{\kappa}_k, \mathrm{d}u^{x}_k)\, \Delta)^{z_{rk}}$$
$$\mathcal{G}\left(\mathrm{d}\xi \,\middle|\, \frac{\alpha_0}{K}\sum_{k=1}^{K} \delta_{(u^{\kappa}_k, u^{x}_k)}, \beta^*\right).$$

From the lemma 2 of [1], we can change the order of integration in $B(\boldsymbol{c}, \boldsymbol{z})$ as

$$B(\boldsymbol{c}, \boldsymbol{z})$$
$$= \left(\int_{\mathcal{M}(\Theta)} g(\boldsymbol{c}, \boldsymbol{z}, \xi)\, \mathcal{G}\left(\mathrm{d}\xi \,\middle|\, \sum_{k=1}^{K} \left(\frac{\alpha_0}{K} + \sum_{r=2}^{R} z_{rk}\right) \delta_{(u^{\kappa}_k, u^{x}_k)}, \beta^*\right)\right)$$
$$\cdot \prod_{r=2}^{R}\prod_{k=1}^{K} \left(\beta^*(u^{x}_k)\, f^{\kappa}(c_r - Fc_{r-1} \mid u^{\kappa}_k)\, f^{x}(x_r \mid u^{x}_k) \left(\sum_{k=1}^{K} \left(\frac{\alpha_0}{K} + \sum_{2 \le j < r} z_{jk} \right) \delta_{(u^{\kappa}_k, u^{x}_k)} \right) (\mathrm{d}u^{\kappa}_k, \mathrm{d}u^{x}_k)\, \Delta \right)^{z_{rk}}.$$

Then, using the relation

$$\prod_{r=2}^{R}\prod_{k=1}^{K}\left(\left(\sum_{k=1}^{K}\left(\frac{\alpha_0}{K}+\sum_{2\le j<r} z_{jk}\right)\delta_{(u_k^\kappa,u_k^x)}\right)(\mathrm{d}u_k^\kappa,\mathrm{d}u_k^x)\right)^{z_{rk}}$$
$$=\int\left(\prod_{r=2}^{R}\prod_{k=1}^{K}P(\mathrm{d}u_k^\kappa,\mathrm{d}u_k^x)^{z_{rk}}\right)\mathcal{DP}\left(\mathrm{d}P\,\middle|\,\frac{\alpha_0}{K}\sum_{k=1}^{K}\delta_{(u_k^\kappa,u_k^x)}\right),$$

$$P\sim\mathcal{DP}\left(\mathrm{d}P\,\middle|\,\frac{\alpha_0}{K}\sum_{k=1}^{K}\delta_{(u_k^\kappa,u_k^x)}\right)\overset{\mathrm{d}}{=}\sum_{k=1}^{K}\pi_k\delta_{(u_k^\kappa,u_k^x)},$$
$$\boldsymbol{\pi}=(\pi_1,\dots,\pi_K)\sim\mathrm{Dir}\left(\frac{\alpha_0}{K},\dots,\frac{\alpha_0}{K}\right), \tag{3}$$

the numerator of (2) becomes

$$\int g(\boldsymbol{c},\boldsymbol{z},\xi)\,p(\boldsymbol{y},\boldsymbol{c},\boldsymbol{z}\mid\xi)\,\mathrm{d}\boldsymbol{c}\,\mathrm{d}\boldsymbol{z}\,\mathcal{G}_K(\mathrm{d}\xi\mid\alpha_0,\beta_0,H^\kappa,\mathbb{u}^x)$$
$$=\exp\left(-\sum_{k=1}^{K}\frac{\alpha_0}{K}\log\left(1+\beta_0T\int_{\mathbb{R}^{d^x}}f^x(x\mid u_k^x)\,\eta(\mathrm{d}x)\right)\right)$$
$$\int\int\left(\int_{\mathcal{M}(\Theta)}g(\boldsymbol{c},\boldsymbol{z},\xi)\,\mathcal{G}\left(\mathrm{d}\xi\,\middle|\,\sum_{k=1}^{K}\left(\frac{\alpha_0}{K}+\sum_{r=2}^{R}z_{rk}\right)\delta_{(u_k^\kappa,u_k^x)},\beta^*\right)\right)$$
$$\cdot\,\mathrm{N}(c_1\mid\mu_{\mathrm{init}}^c,(\Sigma_{\mathrm{init}}^c)^{-1})\prod_{r=2}^{R}\mathrm{N}\left(c_r-Fc_{r-1}\mid 0,V\right)^{\left(1-\sum_{k=1}^{K}z_{rk}\right)}\prod_{r=1}^{R}\mathrm{N}(y_r-Gc_r\mid 0,W)$$
$$\cdot\prod_{r=2}^{R}\prod_{k=1}^{K}\left(\beta^*(u_k^x)\,f^\kappa(c_r-Fc_{r-1}\mid u_k^\kappa)\,f^x(x_r\mid u_k^x)\,\Delta\right)^{z_{rk}}$$
$$\cdot\left(\int\left(\prod_{r=2}^{R}\prod_{k=1}^{K}P(\mathrm{d}u_k^\kappa,\mathrm{d}u_k^x)^{z_{rk}}\right)\mathcal{DP}\left(\mathrm{d}P\,\middle|\,\frac{\alpha_0}{K}\sum_{k=1}^{K}\delta_{(u_k^\kappa,u_k^x)}\right)\right)\mathrm{d}\boldsymbol{c}\,\mathrm{d}\boldsymbol{z}\prod_{k=1}^{K}H^\kappa(\mathrm{d}u_k^\kappa).$$

On the other hand, the denominator of (2) becomes

$$
\begin{aligned}
&\int p(\boldsymbol{y}, \boldsymbol{c}, \boldsymbol{z} \mid \xi)\, \mathcal{G}_K(\mathrm{d}\xi \mid \alpha_0, \beta_0, H^\kappa, \mathbb{u}^x) \\
&\quad = \exp\left(-\sum_{k=1}^{K} \frac{\alpha_0}{K} \log\left(1 + \beta_0 T \int_{\mathbb{R}^{d^x}} f^x(x \mid u_k^x)\, \eta(\mathrm{d}x) \right) \right) \\
&\qquad \int\int \mathrm{N}(c_1 \mid \mu^c_{\text{init}}, (\Sigma^c_{\text{init}})^{-1}) \prod_{r=2}^{R} \mathrm{N}\left(c_r - F c_{r-1} \mid 0, V\right)^{\left(1-\sum_{k=1}^{K} z_{rk}\right)} \prod_{r=1}^{R} \mathrm{N}(y_r - G c_r \mid 0, W) \\
&\qquad\quad \cdot \prod_{r=2}^{R}\prod_{k=1}^{K} \left(\beta^*(u_k^x)\, f^\kappa(c_r - F c_{r-1} \mid u_k^\kappa)\, f^x(x_r \mid u_k^x)\, \Delta\right)^{z_{rk}} \\
&\qquad\quad \cdot \left(\int \left(\prod_{r=2}^{R}\prod_{k=1}^{K} P(\mathrm{d}u_k^\kappa, \mathrm{d}u_k^x)^{z_{rk}} \right) \mathcal{DP}\left(\mathrm{d}P \,\middle|\, \frac{\alpha_0}{K} \sum_{k=1}^{K} \delta_{(u_k^\kappa, u_k^x)} \right) \right) \mathrm{d}\boldsymbol{c}\, \mathrm{d}\boldsymbol{z} \prod_{k=1}^{K} H^\kappa(\mathrm{d}u_k^\kappa).
\end{aligned}
$$

Therefore, (2) can be expressed as

$$
\begin{aligned}
&\frac{\int g(\boldsymbol{c}, \boldsymbol{z}, \xi)\, p(\boldsymbol{y}, \boldsymbol{c}, \boldsymbol{z} \mid \xi) \mathrm{d}\boldsymbol{c}\, \mathrm{d}\boldsymbol{z}\, \mathcal{G}_K(\mathrm{d}\xi \mid \alpha_0, \beta_0, H^\kappa, \mathbb{u}^x)}{\int p(\boldsymbol{y}, \boldsymbol{c}, \boldsymbol{z} \mid \xi)\, \mathrm{d}\boldsymbol{c}\, \mathrm{d}\boldsymbol{z}\, \mathcal{G}_K(\mathrm{d}\xi \mid \alpha_0, \beta_0, H^\kappa, \mathbb{u}^x)} \\
&\qquad = \int g(\boldsymbol{c}, \boldsymbol{z}, \xi)\, \mathcal{G}\left(\mathrm{d}\xi \,\middle|\, \sum_{k=1}^{K} \left(\frac{\alpha_0}{K} + \sum_{r=2}^{R} z_{rk} \right) \delta_{(u_k^\kappa, u_k^x)}, \beta^* \right) p(\mathrm{d}\boldsymbol{c}, \mathrm{d}\boldsymbol{z}, \mathrm{d}\mathbb{u}^\kappa \mid \mathbb{u}^x, \boldsymbol{y}),
\end{aligned}
$$

where $p(\mathrm{d}\boldsymbol{c}, \mathrm{d}\boldsymbol{z}, \mathrm{d}\mathbb{u}^\kappa \mid \mathbb{u}^x, \boldsymbol{y})$ is the posterior of $(\boldsymbol{c}, \boldsymbol{z}, \mathbb{u}^\kappa)$ given $\mathbb{u}^x$ and $\boldsymbol{y}$ that is proportional to

$$
\begin{aligned}
&p(\mathrm{d}\boldsymbol{c}, \mathrm{d}\boldsymbol{z}, \mathrm{d}\mathbb{u}^\kappa \mid \mathbb{u}^x, \boldsymbol{y}) \\
&\propto \mathrm{N}(c_1 \mid \mu^c_{\text{init}}, (\Sigma^c_{\text{init}})^{-1}) \prod_{r=2}^{R} \mathrm{N}\left(c_r - F c_{r-1} \mid 0, V\right)^{\left(1-\sum_{k=1}^{K} z_{rk}\right)} \prod_{r=1}^{R} \mathrm{N}(y_r - G c_r \mid 0, W) \\
&\quad \cdot \prod_{r=2}^{R}\prod_{k=1}^{K} \left(\beta^*(u_k^x)\, f^\kappa(c_r - F c_{r-1} \mid u_k^\kappa)\, f^x(x_r \mid u_k^x)\, \Delta\right)^{z_{rk}} \\
&\quad \cdot \left(\int \left(\prod_{r=2}^{R}\prod_{k=1}^{K} P(\mathrm{d}u_k^\kappa, \mathrm{d}u_k^x)^{z_{rk}} \right) \mathcal{DP}\left(\mathrm{d}P \,\middle|\, \frac{\alpha_0}{K} \sum_{k=1}^{K} \delta_{(u_k^\kappa, u_k^x)} \right) \right) \mathrm{d}\boldsymbol{c}\, \mathrm{d}\boldsymbol{z} \prod_{k=1}^{K} H^\kappa(\mathrm{d}u_k^\kappa) \qquad (4)
\end{aligned}
$$

and this corresponds to (1).

Next, consider a hidden variable π to indicate the weights of the random measure P in (3) distributes to the Dirichlet process $\mathcal{DP}(\cdot)$. Using $\boldsymbol{\pi}$, we can express the integration respect to

Box 1: Estimation steps of our method.

Initialize variational posterior q and hyperparameters.
repeat
    update $q(\boldsymbol{c})$.
    update $q(\boldsymbol{z})$.
    update $q(\boldsymbol{\pi})$.
    update $q(\mathbb{u}^{\kappa})$.
    update $\mathbb{u}^{x}$.
    update $\mu^{c}_{\text{init}}, \Sigma^{c}_{\text{init}}$.
    update F, V.
    update G, W.
    update α_0.
    merge similar components.
until the evidence lower bound $\mathcal{L}(q)$ converges

$\mathcal{DP}(\cdot)$ as the integration respect to $\boldsymbol{\pi}$:

$$\int \left(\prod_{r=2}^{R} \prod_{k=1}^{K} P(\mathrm{d}u_k^{\kappa}, \mathrm{d}u_k^{x})^{z_{rk}} \right) \mathcal{DP} \left(\mathrm{d}P \,\middle|\, \frac{\alpha_0}{K} \sum_{k=1}^{K} \delta_{(u_k^{\kappa}, u_k^{x})} \right)$$
$$= \int_{S^{K-1}} \prod_{r=2}^{R} \prod_{k=1}^{K} \pi_k^{z_{rk}} \, \mathrm{Dir} \left(\boldsymbol{\pi} \,\middle|\, \frac{\alpha_0}{K}, \ldots, \frac{\alpha_0}{K} \right) \mathrm{d}\boldsymbol{\pi}.$$

Substituting this relation into (4), we obtain the second statement. □

3 Details of estimation steps

Box 1 shows the entire flow of the estimation procedure in our method. We give the details of how to update hidden variables and hyperparameters in the estimation step. We also explain some heuristic techniques for obtaining good variational posterior.

3.1 Update formula

The complete log likelihood of our model is expressed as

$$
\begin{aligned}
&\log p(\boldsymbol{y}, \boldsymbol{c}, \boldsymbol{z}, \boldsymbol{\pi}, \mathbb{u}^\kappa \mid \mathbb{u}^x) \\
&= \log \mathrm{N}(c_1 \mid \mu^c_{\text{init}}, \Lambda^c_{\text{init}}) + \sum_{r=2}^{R} \left(1 - \sum_{k=1}^{K} z_{rk}\right) \log \mathrm{N}\left(c_r - F c_{r-1} \mid 0, V\right) + \sum_{r=1}^{R} \log \mathrm{N}(y_r - G c_r \mid 0, W) \\
&\quad + \sum_{r=2}^{R} \sum_{k=1}^{K} z_{rk} \left(\log \beta_0 + \log \mathrm{N}\left(c_r - F c_{r-1} \mid \mu^\kappa_k, \Lambda^\kappa_k\right) + \log f^x(x_r \mid u^x_k) + \log \Delta\right) \\
&\quad - \sum_{k=1}^{K} \left(\frac{\alpha_0}{K} + \sum_{r=2}^{R} z_{rk}\right) \log \left(1 + \beta_0 T \int_{\mathbb{R}^{d^x}} f^x(x \mid u^x_k)\, \eta(\mathrm{d}x)\right) \\
&\quad + \sum_{r=2}^{R} \sum_{k=1}^{K} z_{rk} \log \pi_k + \log \mathrm{Dir}\left(\boldsymbol{\pi} \,\middle|\, \frac{\alpha_0}{K}, \ldots, \frac{\alpha_0}{K}\right) + \sum_{k=1}^{K} \log \mathrm{NW}(\mu^\kappa_k, \Lambda^\kappa_k \mid m^\kappa, \gamma^\kappa, \nu^\kappa, S^\kappa) \\
&\quad + \text{const.}
\end{aligned}
$$

Here, we substitute Gaussian kernel $\mathrm{N}(\cdot \mid \mu^\kappa_k, \Lambda^\kappa_k)$ for $f^\kappa(\cdot \mid u^\kappa_k)$ in the loglikelihood.

Under the independent constraint, the variational posterior of each hidden variable maximizing the evidence lower bound $\mathcal{L}(q)$ can be obtained by calculating the expectation of the complete loglikelihood with respect to other hidden variables:

$$
\begin{aligned}
q(\boldsymbol{c}) &\propto \exp\left(\mathrm{E}_{q(\boldsymbol{z}, \boldsymbol{\pi}, \mathbb{u}^\kappa)}\left[\log p(\boldsymbol{y}, \boldsymbol{c}, \boldsymbol{z}, \boldsymbol{\pi}, \mathbb{u}^\kappa \mid \mathbb{u}^x)\right]\right), \\
q(\boldsymbol{z}) &\propto \exp\left(\mathrm{E}_{q(\boldsymbol{c}, \boldsymbol{\pi}, \mathbb{u}^\kappa)}\left[\log p(\boldsymbol{y}, \boldsymbol{c}, \boldsymbol{z}, \boldsymbol{\pi}, \mathbb{u}^\kappa \mid \mathbb{u}^x)\right]\right), \\
q(\boldsymbol{\pi}) &\propto \exp\left(\mathrm{E}_{q(\boldsymbol{c}, \boldsymbol{z}, \mathbb{u}^\kappa)}\left[\log p(\boldsymbol{y}, \boldsymbol{c}, \boldsymbol{z}, \boldsymbol{\pi}, \mathbb{u}^\kappa \mid \mathbb{u}^x)\right]\right), \\
q(\mathbb{u}^\kappa) &\propto \exp\left(\mathrm{E}_{q(\boldsymbol{c}, \boldsymbol{z}, \boldsymbol{\pi})}\left[\log p(\boldsymbol{y}, \boldsymbol{c}, \boldsymbol{z}, \boldsymbol{\pi}, \mathbb{u}^\kappa \mid \mathbb{u}^x)\right]\right).
\end{aligned}
$$

Left side of these equations reduce to closed form so that we can update each posterior without functional optimization. Hereafter, we show update formulas for these variables.

Update $q(\boldsymbol{c})$

We define new variables as

$$V_r = \sum_{k=1}^{K} \mathrm{E}\left[z_{rk}\right] \mathrm{E}\left[\Lambda_k^{\kappa}\right] + \left(1 - \sum_{k=1}^{K} \mathrm{E}\left[z_{rk}\right]\right) V, \qquad r = 2, \ldots, R,$$
$$o_r = V_r^{-1} \left(\sum_{k=1}^{K} \mathrm{E}\left[z_{rk}\right] \mathrm{E}\left[\Lambda_k^{\kappa} \mu_k^{\kappa}\right]\right), \qquad r = 2, \ldots, R.$$

Then, the distribution of $\boldsymbol{c}$ that maximizes $\mathcal{L}(q)$ coincides with the posterior of the following linear state space model:

$$\begin{aligned} c_1 &\sim \mathrm{N}(\mu_{\text{init}}^c, (\Sigma_{\text{init}}^c)^{-1}), \\ c_r &= F c_{r-1} + o_r + v_r, \quad && v_r \sim \mathrm{N}(0, V_r), \quad r = 2, \ldots, R \\ y_r &= G c_r + w_r, && w_r \sim \mathrm{N}(0, W), \quad r = 1, \ldots, R. \end{aligned} \tag{5}$$

The posterior of this linear state space model becomes Gaussian distribution

$$\mathrm{N}\left(\boldsymbol{c} \mid \{\mu_r^c\}_{r=1}^R, \{\Sigma_r^c\}_{r=1}^R, \{\Sigma_{r-1,r}^c\}_{r=2}^R\right)$$

that indexed by mean vectors $\{\mu_r^c\}_{r=1}^R$, covariance matrices $\{\Sigma_r^c\}_{r=1}^R$ and pairwise covariance matrices $\{\Sigma_{r-1,r}^c\}_{r=2}^R$. These parameters can be calculated by the Kalman smoother.

Therefore, we apply the Kalman smoother to calculate $(\{\mu_r^c\}_{r=1}^R, \{\Sigma_r^c\}_{r=1}^R, \{\Sigma_{r-1,r}^c\}_{r=2}^R)$ under the state space model (5) and update $q(\boldsymbol{c})$ as

$$q(\boldsymbol{c}) \leftarrow \mathrm{N}\left(\boldsymbol{c} \mid \{\mu_r^c\}_{r=1}^R, \{\Sigma_r^c\}_{r=1}^R, \{\Sigma_{r-1,r}^c\}_{r=2}^R\right).$$

The most computationally heavy part of our method is this update. The run-time for this Kalman smoother is about $O(R((d^{\kappa})^3 + d^y d^{\kappa}))$ and the required memory is about $O(R(d^{\kappa})^2)$.

Update $q(\boldsymbol{z})$

We define new variables as

$$
\begin{aligned}
\rho_{rk} &= \log\beta_0 + \mathrm{E}\left[\log \mathrm{N}\left(c_r - Fc_{r-1} \mid \mu_k^\kappa, \Lambda_k^\kappa\right)\right] + \log f^x(x_r \mid u_k^x) + \log\Delta, \\
&\qquad - \log\left(1 + \beta_0 T \int_{\mathbb{R}^{d^x}} f^x(x \mid u_k^x)\,\eta(\mathrm{d}x)\right) + \mathrm{E}\left[\log\pi_k\right], \quad r = 2,\ldots,R,\ k = 1,\ldots,K \\
\rho_{r0} &= \mathrm{E}\left[\log \mathrm{N}\left(c_r - Fc_{r-1} \mid 0, V\right)\right], \quad r = 2,\ldots,R.
\end{aligned}
$$

Using these variables, we update $q(\boldsymbol{z})$ as

$$
q(\boldsymbol{z}) \leftarrow \prod_{r=2}^{R}\left(1 - \sum_{k=1}^{K}\zeta_{rk}\right)^{\left(1-\sum_{k=1}^{K} z_{rk}\right)} \prod_{k=1}^{K}\zeta_{rk}^{z_{rk}},
$$

where

$$
\zeta_{rk} = \frac{\exp(\rho_{rk})}{\sum_{k=0}^{K}\exp(\rho_{rk})}, \quad r = 2,\ldots,R,\ k = 1,\ldots,K.
$$

Update $q(\boldsymbol{\pi})$

We update $q(\boldsymbol{\pi})$ as

$$
q(\boldsymbol{\pi}) \leftarrow \mathrm{Dir}\left(\boldsymbol{\pi} \mid \alpha_1,\ldots,\alpha_K\right), \tag{6}
$$

where

$$
\alpha_k = \frac{\alpha_0}{K} + \sum_{r=2}^{R}\mathrm{E}\left[z_{rk}\right], \quad k = 1,\ldots,K.
$$

Update $q(\mathbb{u}^\kappa)$

We update $q(u_k^\kappa)$, $k = 1,\ldots,K$ respectively as

$$
q(u_k^\kappa) \leftarrow \mathrm{NW}(\mu_k^\kappa, \Lambda_k^\kappa \mid m_k^\kappa, \gamma_k^\kappa, \nu_k^\kappa, S_k^\kappa), \tag{7}
$$

where

$$
\begin{aligned}
m_k^\kappa &= \frac{\gamma^\kappa m^\kappa + \sum_{r=2}^R \mathrm{E}\left[z_{rk}\right] \mathrm{E}\left[c_r - F c_{r-1}\right]}{\gamma^\kappa + \sum_{r=2}^R \mathrm{E}\left[z_{rk}\right]}, \\
\gamma_k^\kappa &= \gamma^\kappa + \sum_{r=2}^R \mathrm{E}\left[z_{rk}\right], \\
\nu_k^\kappa &= \nu^\kappa + \sum_{r=2}^R \mathrm{E}\left[z_{rk}\right], \\
S_k^\kappa &= \left((S^\kappa)^{-1} + \sum_{r=2}^R \mathrm{E}\left[z_{rk}\right] \mathrm{E}\left[(c_r - F c_{r-1})(c_r - F c_{r-1})^{\mathrm{T}} + \gamma^\kappa m^\kappa m^{\kappa \mathrm{T}} - \gamma_k^\kappa m_k^\kappa m_k^{\kappa \mathrm{T}} \right] \right)^{-1}.
\end{aligned}
$$

Update $\mathbb{u}^x$

The term depends on u_k^x in $\mathcal{L}(q)$ is

$$
\sum_{r=2}^R \mathrm{E}\left[z_{rk}\right] \log f^x(x_r \mid u_k^x) - \left(\frac{\alpha_0}{K} + \sum_{r=2}^R \mathrm{E}\left[z_{rk}\right] \right) \log \left(1 + \beta_0 T \int_{\mathbb{R}^{d^x}} f^x(x \mid u_k^x)\, \eta(\mathrm{d}x) \right).
$$

We find u_k^x that maximizes $\mathcal{L}(q)$ using nonlinear optimization and update u_k^x by this value.

Update $\mu_{\mathrm{init}}^c, \Sigma_{\mathrm{init}}^c$

We update the initial state mean μ_{init}^c and the initial state covariance matrix Σ_{init}^c as

$$
\begin{aligned}
\mu_{\mathrm{init}}^c &\leftarrow \mathrm{E}\left[c_1\right], \\
\Sigma_{\mathrm{init}}^c &\leftarrow \mathrm{E}\left[c_1 c_1^{\mathrm{T}}\right] - \mathrm{E}\left[c_1\right] \mathrm{E}\left[c_1\right]^{\mathrm{T}}.
\end{aligned}
$$

Update F, V

We define Ψ and Ω as

$$
\begin{aligned}
\Psi &= \sum_{r=2}^R \left(\sum_{k=1}^K \mathrm{E}\left[z_{rk}\right] \mathrm{E}\left[\Lambda_k\right] + \sum_{k=1}^K \left(1 - \mathrm{E}\left[z_{rk}\right]\right) V \right) \otimes \mathrm{E}\left[c_{r-1} c_{r-1}^{\mathrm{T}}\right], \\
\Omega &= \sum_{r=2}^R \left(\sum_{k=1}^K \mathrm{E}\left[z_{rk}\right] \mathrm{E}\left[\Lambda_k (c_r - \mu_k) c_{r-1}^{\mathrm{T}}\right] + \left(1 - \sum_{k=1}^K \mathrm{E}\left[z_{rk}\right] \right) V \mathrm{E}\left[c_r c_{r-1}^{\mathrm{T}}\right] \right)
\end{aligned}
$$

and solve the following matrix equation

$$\Psi\phi = \mathrm{vec}(\Omega).$$

Here, $\mathrm{vec}(\cdot)$ is a vectorization operator and $\mathrm{vec}(\Omega)$ corresponds to the vector obtained by stacking the rows of Ω. Denote the solution of this equation as ϕ. Using this ϕ, we update F as

$$F \leftarrow \begin{pmatrix} \phi_1 & \phi_2 & \cdots & \phi_{d^\kappa} \\ \phi_{d^\kappa+1} & \phi_{d^\kappa+2} & \cdots & \phi_{2d^\kappa} \\ \vdots & \vdots & \ldots & \vdots \\ \phi_{(d^\kappa-1)d^\kappa+1} & \phi_{(d^\kappa-1)d^\kappa+2} & \cdots & \phi_{d^{\kappa 2}} \end{pmatrix}.$$

where ϕ_j is a j-th element of ϕ. After updating F, we update V as

$$V \leftarrow \left(\frac{1}{R - 1 - \sum_{r=2}^{R}\sum_{k=1}^{K} \mathrm{E}\left[z_{rk}\right]} \sum_{r=2}^{R}\sum_{k=1}^{K} \mathrm{E}\left[z_{rk}\right] \mathrm{E}\left[\left(c_r - Fc_{r-1}\right)\left(c_r - Fc_{r-1}\right)^{\mathrm{T}} \right] \right)^{-1}.$$

This update gives the maximum optimum of $\mathcal{L}(q)$ with respect to F and V. However, this approach is sensitive to contamination and misspecification of the initial value for q. Consider that a spike occurred between the $(r-1)$-th frame and the r-th frame even though the estimated spike occurrence probability $\sum_{k=1}^{K} \mathrm{E}[z_{rk}]$ is small. Then, the rapid fraction in the movie due to this spike may cause the precision matrix V to be underestimated. In our method, we have to select an appropriate initial value for $q(\boldsymbol{z})$. If this value fails to express the spiking activities, then contamination would occur, and the variational posterior would differ from the true posterior.

One possible solution for this problem is to obtain robust approximation close to the maximum optimum. In the rest of this section, we explain an alternative update using a modified Multivariate Least Trimmed Squared (MLTS) estimator.

The MLTS estimator is a highly robust estimator used in multivariate regression [2]. Let $\mathcal{H}$ be the subset of $\{2, \ldots, R\} \setminus \mathcal{R}(q(\boldsymbol{z}))$ where $\mathcal{R}(q(\boldsymbol{z})) = \{r \mid \sum_{k=1}^{K} \mathrm{E}[z_{rk}] > \epsilon\}$ for sufficiently

small $\epsilon > 0$. We define $(F(\mathcal{H}), V(\mathcal{H}))$ as

$$F(\mathcal{H}) = \left(\sum_{r\in\mathcal{H}} \mathrm{E}\left[c_r c_{r-1}^{\mathrm{T}}\right]\right)\left(\sum_{r\in\mathcal{H}} \mathrm{E}\left[c_{r-1} c_{r-1}^{\mathrm{T}}\right]\right),$$

$$V(\mathcal{H}) = \left(\frac{C}{h}\sum_{r\in\mathcal{H}} \mathrm{E}\left[\left(c_r - F(\mathcal{H})c_{r-1}\right)\left(c_r - F(\mathcal{H})c_{r-1}\right)^{\mathrm{T}}\right]\right)^{-1}$$

for two parameters h and C. Using these variables, MLTS estimator $(F_{\mathrm{MLTS}}, V_{\mathrm{MLTS}})$ is defined as

$$(F_{\mathrm{MLTS}}, V_{\mathrm{MLTS}}) = \left(F(\hat{\mathcal{H}}), \frac{1}{C}V(\hat{\mathcal{H}})\right),$$

$$\hat{\mathcal{H}} = \underset{\{\mathcal{H}|\#\mathcal{H}=h\}}{\arg\max}\ \det(V(\mathcal{H})).$$

Here, h is a cardinality of $\mathcal{H}$ and C is a correction factor for maintaining consistency. This MLTS estimator is also used for outlier detection. Define the Mahalanobis distance s_r, $r = 2, \ldots, R$ as

$$s_r = \mathrm{E}\left[(c_r - F_{\mathrm{MLTS}}c_{r-1})^{\mathrm{T}} V_{\mathrm{MLTS}} (c_r - F_{\mathrm{MLTS}}c_{r-1})\right], \quad r = 2, \ldots, R.$$

If s_r takes a large value, then the large fraction may occur in the movie between the $(r-1)$-th frame and the r-th frame; it suggests the existence of spikes at this frame. Using this s_r, another version of the MLTS estimator called the reweighted MLTS estimator $(F_{\mathrm{rMLTS}}, V_{\mathrm{rMLTS}})$ is defined as

$$(F_{\mathrm{rMLTS}}, V_{\mathrm{rMLTS}}) = (F(\tilde{\mathcal{H}}), V(\tilde{\mathcal{H}})),$$

$$\tilde{\mathcal{H}} = \{r \mid s_r < \chi^2_{d^\kappa}(\alpha),\ r \in \{2, \ldots, R\} \setminus \mathcal{R}(q(\boldsymbol{z}))\},$$

where $\chi^2_{d^\kappa}(\alpha)$ is the upper α% point of the chi-squared distribution whose degree of freedom is d^κ.

A disadvantage of the MLTS estimator is that it involves a large computational cost. To calculate the exact MLTS estimator requires calculating the above equation $\binom{R-\#\mathcal{R}(q(\boldsymbol{z}))}{h}$ times. Therefore, in our method, we use the MLTS estimator only for setting the initial value and use

the former estimator in the following iterations.

Update G, W

We update the observation matrix G as

$$G \leftarrow \left(\sum_{r=1}^{R} y_r \mathrm{E}\left[c_r\right]^{\mathrm{T}}\right)\left(\sum_{r=1}^{R} \mathrm{E}\left[c_r c_r^{\mathrm{T}}\right]\right)^{-1}.$$

It is also able to update the observation precision matrix W in the closed form. Because we restrict W as a diagonal matrix, the required computational cost for the update is linear to the dimension of the movie d^y, not quadratic.

First, we apply the Cholesky decomposition to the sum of the posterior mean of $c_r c_r^{\mathrm{T}}$, $r = 1, \ldots, R$ as

$$\sum_{r=1}^{R} \mathrm{E}\left[c_r c_r^{\mathrm{T}}\right] = LL^{\mathrm{T}}.$$

Next, using this L, we define w_i, $i = 1, \ldots, d^y$ as

$$w_i = \left(\frac{1}{R}\left(\sum_{r=1}^{R} y_{ri}^2 - 2\sum_{r=1}^{R} y_{ri}\left(G\mathrm{E}\left[c_r\right]\right)_i + \sum_{j=1}^{d^\kappa}(GL)_{ij}^2\right)\right)^{-1}, \quad i = 1, \ldots, d^y$$

where $(\cdot)_i$ is a i-th element in a vector and $(\cdot)_{ij}$ is a (i, j)-th element in a matrix. Finally, we update W as

$$W \leftarrow \begin{pmatrix} w_1 & & & \\ & w_2 & & \\ & & \ddots & \\ & & & w_{d^y} \end{pmatrix}.$$

Update α_0

The term that depends on α_0 in $\mathcal{L}(q)$ is

$$\log\Gamma(\alpha_0) - K\log\Gamma\left(\frac{\alpha_0}{K}\right) + \frac{\alpha_0}{K}\sum_{k=1}^{K}\left(\mathrm{E}\left[\log\pi_k\right] - \log\left(1 + \beta_0 T\int_{\mathbb{R}^{d^x}} f^x(x \mid u_k^x)\,\eta(\mathrm{d}x)\right)\right)$$

We find that α_0 maximizes this term using nonlinear optimization, and update α_0 by this value.

3.2 Posterior mean of hidden variables and its implication

In our model, the posterior expectation of each parameter gives the deconvolution results. For example, $\mathrm{E}[G\mu_k^\kappa]$ is the shape of the k-th neuron and $\mathrm{E}[Gc_r]$, $r = 1, \ldots, R$ is the denoised movie taking account of non-linearity that occurred from the spiking activities. Box 2 shows some implications for the posterior mean of variables in our model.

Box 2: Implication for variational posterior mean and hyper parameters.

Implication	Posterior mean
Cell shape of k-th neuron	$G\,\mathrm{E}\left[\mu_k^\kappa\right]$
Spike occurrence time of k-th neuron	$\left\{ r \,\middle\vert\, \arg\max_{k'} \mathrm{E}\left[z_{rk'}\right] = k,\ \sum_{k=1}^{K} \mathrm{E}\left[z_{rk}\right] > \epsilon \right\},$ for sufficiently small $\epsilon > 0$
Receptive field of k-th neuron	$\left\{x \mid f^x(x \mid u_k^x) > \epsilon\right\},$ for some $\epsilon > 0$
Denoised movie	$G\,\mathrm{E}\left[c_r\right],\ r = 1, \ldots, R$
Intensity function	$\mathrm{E}\left[\lambda(\kappa, x \mid \xi)\right]$

In the estimation step, we also have to calculate the posterior mean of hidden variables to update the variational posterior. Our generative model definition and the independence constraint reduce these expectations to the closed form. The following equations show the posterior mean of hidden variables that appeared in the update formulas.

$$
\begin{aligned}
\mathrm{E}\left[c_r - Fc_{r-1}\right] &= \mu^c_r - F\mu^c_{r-1}, \\
\mathrm{E}\left[c_r c_r^{\mathrm{T}}\right] &= \mu^c_r \mu^{c\mathrm{T}}_r + \Sigma^c_r, \\
\mathrm{E}\left[c_{r-1} c_r^{\mathrm{T}}\right] &= \mu^c_{r-1} \mu^{c\mathrm{T}}_r + \Sigma^c_{r-1,r}, \\
\mathrm{E}\left[(c_r - Fc_{r-1})(c_r - Fc_{r-1})^{\mathrm{T}}\right] &= (\mu^c_r - F\mu^c_{r-1})(\mu^c_r - F\mu^c_{r-1})^{\mathrm{T}} \\
&\quad + \Sigma^c_r - F\Sigma^c_{r-1,r} - \Sigma^c_{r,r-1}F^{\mathrm{T}} + F\Sigma^c_{r-1}F^{\mathrm{T}}, \\
\mathrm{E}\left[\log \mathrm{N}(c_r - Fc_{r-1} \mid \mu^\kappa_k, \Lambda^\kappa_k)\right] &= -\frac{d^\kappa}{2}\log 2\pi + \frac{1}{2}\sum_{j=1}^{d^\kappa} \psi\left(\frac{\nu^\kappa_k + 1 - j}{2}\right) + \frac{d^\kappa}{2}\log 2 + \frac{1}{2}\log|S^\kappa_k| \\
&\quad - \frac{1}{2}(\mu^c_r - F\mu^c_{r-1} - m^\kappa_k)^{\mathrm{T}} S^\kappa_f (\mu^c_r - F\mu^c_{r-1} - m^\kappa_k) - \frac{d^\kappa}{2\gamma^\kappa_k} \\
&\quad - \frac{\nu^\kappa_k}{2}\mathrm{tr}\left(S^\kappa_k\left(\Sigma^c_r - F\Sigma^c_{r-1,r} - \Sigma^c_{r,r-1}F^{\mathrm{T}} + F\Sigma^c_{r-1}F^{\mathrm{T}}\right)\right), \\
\mathrm{E}\left[\log \mathrm{N}(c_r - Fc_{r-1} \mid 0, V)\right] &= -\frac{d^\kappa}{2}\log 2\pi + \frac{1}{2}\log|V| - \frac{1}{2}(\mu^c_r - F\mu^c_{r-1})^{\mathrm{T}} V (\mu^c_r - F\mu^c_{r-1}) \\
&\quad - \frac{1}{2}\mathrm{tr}\left(V\left(\Sigma^c_r - F\Sigma^c_{r-1,r} - \Sigma^c_{r,r-1}F^{\mathrm{T}} + F\Sigma^c_{r-1}F^{\mathrm{T}}\right)\right), \\
\mathrm{E}\left[z_{rk}\right] &= \zeta_{rk}, \\
\mathrm{E}\left[\log \pi_k\right] &= \psi(\alpha_k) - \psi\left(\sum_{k=1}^{K}\alpha_k\right), \\
\mathrm{E}\left[\Lambda^\kappa_k \mu^\kappa_k\right] &= \nu^\kappa_k S^\kappa_k m^\kappa_k, \\
\mathrm{E}\left[\Lambda^\kappa_k\right] &= \nu^\kappa_k S^\kappa_k, \\
\mathrm{E}\left[\mathrm{N}(\kappa \mid \mu^\kappa_k, \Lambda^\kappa_k)\right] &= \mathrm{St}\left(\kappa \,\middle|\, m^\kappa_k, \frac{(\nu^\kappa_k + 1 - d^\kappa)\gamma^\kappa_k}{1 + \gamma^\kappa_k} S^\kappa_k, \nu^\kappa_k + 1 - d^\kappa\right).
\end{aligned}
$$

Here, ψ is the digamma function.

At the end of this section, we show how to calculate the posterior mean of the intensity function $\lambda(\kappa, x \mid \xi)$. Given other variables, ξ distributes to

$$
\mathcal{G}\left(\mathrm{d}\xi \,\middle|\, \sum_{k=1}^{K}\left(\frac{\alpha_0}{K} + \sum_{r=2}^{R} z_{rk}\right)\delta_{(u^\kappa_k, u^x_k)}, \beta^*\right)
$$

and this ξ is also expressed as

$$\xi = \sum_{k=1}^{K} \frac{\beta_0 g_k}{1 + \beta_0 T \int f^x(x \mid u_k^x)\eta(\mathrm{d}x)} \delta_{(u_k^\kappa, u_k^x)},$$

$$g_k \sim \mathrm{Gamma}\left(\frac{\alpha_0}{K} + \sum_{r=2}^{R} z_{rk}\right).$$

Therefore, posterior mean of $\lambda(\kappa, x \mid \xi)$ is calculated as

$$\begin{aligned}
&\mathrm{E}\left[\lambda(\kappa, x \mid \xi)\right] \\
&= \sum_{k=1}^{K} \frac{\beta_0\left(\frac{\alpha_0}{K} + \sum_{r=2}^{R} \zeta_{rk}\right)}{1 + \beta_0 T \int f^x(x \mid u_k^x)\eta(\mathrm{d}x)} \mathrm{St}\left(\kappa \,\middle|\, m_k^\kappa, \frac{(\nu_k^\kappa + 1 - d^\kappa)\gamma_k^\kappa}{1 + \gamma_k^\kappa} S_k^\kappa, \nu_k^\kappa + 1 - d^\kappa\right) f^x(x \mid u_k^x).
\end{aligned}$$

This equation also shows that the tuning curve of the k-th neuron is expressed as

$$\frac{\beta_0\left(\frac{\alpha_0}{K} + \sum_{r=2}^{R} \zeta_{rk}\right)}{1 + \beta_0 T \int f^x(x \mid u_k^x)\eta(\mathrm{d}x)} f^x(x \mid u_k^x).$$

3.3 Initialization

Because the evidence lower bound $\mathcal{L}(q)$ is not convex, the optimization problem does not have a global optimum. Hence, the solution obtained in the estimation step is nothing but a local optimum, and it depends strongly on the initial value.

To obtain a good variational posterior, it is important to decide a good initial value beforehand. The following procedure is an example of how to decide the initial value.

1. Decide K and (α_0, β_0) as appropriate values.

2. Subtract an average from movie vector y_r as

$$y_r \leftarrow y_r - \frac{1}{R} \sum_{r'=1}^{R} y_{r'}, \quad r = 1, \ldots, R$$

3. Apply principal components analysis to reduce the dimension of y_r from d^y to d^κ. Define c_r, $r = 1, \ldots, R$ as these transformed vectors and G as the transpose of the transformation matrix.

4. Apply the reweighted MLTS algorithm for c_r, $r = 1, \ldots, R$ to obtain (F, V). Define $\mathcal{R}$ be the time indices not used in the reweighted MLTS algorithm and define o_r, $r = 2, \ldots, R$ as

$$o_r = \begin{cases} c_r - Fc_{r-1}, & (\text{if } r \notin \mathcal{R}), \\ 0, & (\text{if } r \in \mathcal{R}). \end{cases}$$

5. Consider the following linear state space model

$$\begin{aligned} c_1 &\sim \mathrm{N}(\mu^c_{\text{init}}, (\Sigma^c_{\text{init}})^{-1}), \\ c_r &= Fc_{r-1} + o_r + u_r, \quad u_r \sim \mathrm{N}(0, V), \quad r = 2, \ldots, R, \\ y_r &= Gc_r + w_r, \quad w_r \sim \mathrm{N}(0, W), \quad r = 1, \ldots, R \end{aligned}$$

and apply Expectation Maximization algorithm to estimate $(\mu^c_{\text{init}}, \Sigma^c_{\text{init}}, W)$. Using estimated $(\mu^c_{\text{init}}, \Sigma^c_{\text{init}}, W)$, calculate the posterior of c_r, $r = 1, \ldots, R$ as

$$\mathrm{N}\left(\boldsymbol{c} \mid \{\mu^c_r\}^R_{r=1}, \{\Sigma^c_r\}^R_{r=1}, \{\Sigma^c_{r-1,r}\}^R_{r=2}\right)$$

by the Kalman smoother.

6. From $\{o_r\}_{r \in \mathcal{R}}$, calculate a sample mean μ and a sample covariance matrix Σ. Using these values, define hyperparameters $(m^\kappa, \gamma^\kappa, \nu^\kappa, S^\kappa)$ as

$$m^\kappa = \mu, \qquad \gamma^\kappa = 1, \qquad \nu^\kappa = d^\kappa, \qquad S^\kappa = \frac{1}{d^\kappa}\Sigma^{-1}.$$

7. Consider that $\{o_r\}_{r \in \mathcal{R}}$ is generated from Gaussian mixture model with K components defined on $\mathbb{R}^{d^\kappa}$ and estimate the parameters $(c^\kappa_k, \mu^\kappa_k, \Lambda^\kappa_k)$, $k = 1, \ldots, K$ using Expectation Maximization algorithm. Here, c^κ_k is a weight of the k-th component, μ^κ_k is a mean vector and Λ^κ_k is a precision matrix. Then, using estimated Gaussian mixture model, divide indices $\mathcal{R}$ into K clusters $\mathcal{R}_1, \ldots, \mathcal{R}_K$ and define ζ_{rk} as

$$\zeta_{rk} = \begin{cases} \frac{c_k \mathrm{N}(o_r | \mu^\kappa_k, \Lambda^\kappa_k)}{\sum_{j=1}^K c_j \mathrm{N}(o_r | \mu^\kappa_j, \Lambda^\kappa_j)}, & (\text{if } r \in \mathcal{R}_k), \\ 0, & (\text{otherwise}). \end{cases}$$

8. Define $(m_k^\kappa, \gamma_k^\kappa, \nu_k^\kappa, S_k^\kappa)$, $k = 1, \ldots, K$ as

$$m_k^\kappa = \mu_k^\kappa, \qquad \gamma_k^\kappa = 1, \qquad \nu_k^\kappa = d^\kappa, \qquad S_k^\kappa = S^\kappa, \qquad k = 1, \ldots, K.$$

9. Consider that x_r, $r \in \mathcal{R}_k$ is generated from a distribution whose density is $f^x(\cdot \mid u_k^x)$ independently and estimate the parameter u_k^x using maximum likelihood estimation. Then, set u_k^x by this estimated value.

10. Define α_k, $k = 1, \ldots, K$ as (6).

3.4 Merging components

Our method assumes that each neuron always emits similar fluorescent footprints in the movie when it spikes. Therefore, if two spikes have similar fluorescence shapes but different luminescence levels, our method may mistakenly regard that these spikes as being generated from different neurons even if there are emitted from same neuron. Such misassignment causes overestimation of the number of neurons.

To avoid such a problem, we perform a merging procedure at each iteration along the evidence lower bound maximization. Although this merging procedure does not always increase the evidence lower bound, it enable us to obtain a good local optimum.

The following are the details of this merging procedure.

1. Define the set $\mathcal{R}_k$, $k = 1, \ldots, K$ as

$$\mathcal{R}_k = \left\{ r \,\middle|\, \arg\max_{k'} \mathrm{E}\left[z_{rk'}\right] = k, \quad \sum_{k=1}^{K} \mathrm{E}\left[z_{rk}\right] > \epsilon \right\},$$

   where ϵ is a sufficient small positive value. This $\mathcal{R}_k$ indicates the estimated spike occurrence indices of k-th neuron.

2. Choose components that satisfy $\#\mathcal{R}_k \geq 1$ and calculate the distance of m_k^κ for all two combinations in the selected components. For example, the angle between two vectors can be used for this distance.

3. Decide a threshold value and select the pair whose distance does not exceed this threshold. Let i be the component of this pair with the larger number of spikes allocated, and let j

be the other component. Then, update ζ_{ri}, ζ_{rj}, $r = 2, \ldots R$, which are the parameters of $q(\boldsymbol{z})$, as

$$\zeta_{ri} \leftarrow \zeta_{ri} + \zeta_{rj},$$
$$\zeta_{rj} \leftarrow 0.$$

Given new $q(\boldsymbol{z})$, also update $(m_i^\kappa, \gamma_i^\kappa, \nu_i^\kappa, S_i^\kappa)$ and $(m_j^\kappa, \gamma_j^\kappa, \nu_j^\kappa, S_j^\kappa)$ using (7).

4. Given new $q(\boldsymbol{z})$, update α_k, $k = 1, \ldots, K$ using (6).

3.5 Removing motion artifact

Our model sometimes detects false positive components due to brain motion artifacts occurred in the experiment. Fig 1 are typical examples. CNMF imposes a regularization constraint in the spatial domain along the optimization and also performs a discarding procedure to remove components whose estimated shape is different from a general cell shape. Conversely, our method does not impose any constraints in the spatial domain, and it may regard rapid fractions in the movie as spikes. Estimating motion artifacts as neurons activities is due to model misspecification error; definitions of our generative model cannot distinguish them. Compo-

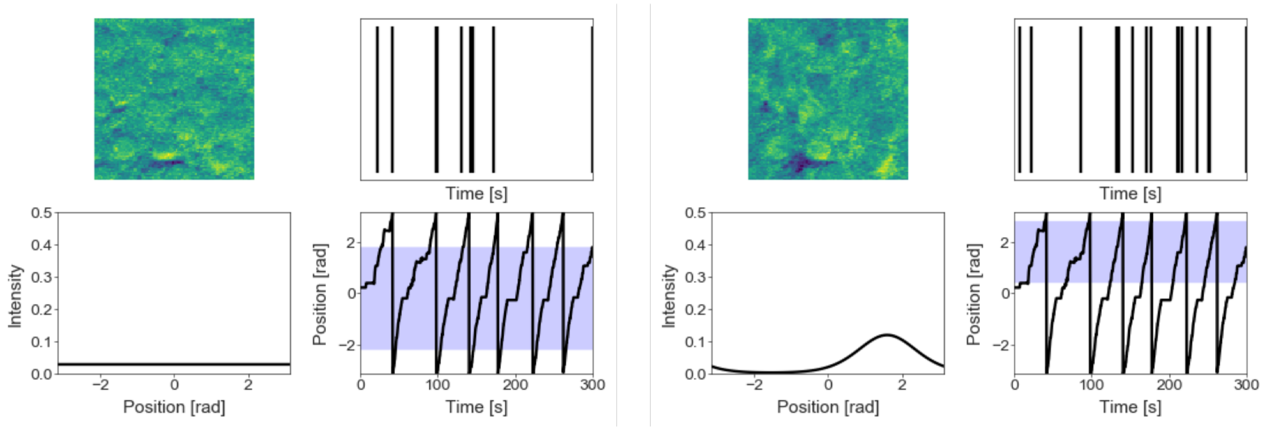

Fig 1: False positive components detected by our method.

nents corresponds to motion artifacts are obviously distinguishable. For example, pixel values distribution of a true component has right heavy tail. Hence, calculating skewness from pixel values enable us to remove such components.

4 Application to spiking activities with constant rate

Our model assumes that spiking activities are modulated by some covariates. However, there may be some neurons whose activities are independent from the focusing covariates and have the constant firing rate along the experiment. Our model is also applicable in such case since the kernel function used for expressing the tuning curve in our model can be uniform distribution. For example, von Mises kernel used in Section 2.2 takes a constant value independent to stimulus when $\tau_x \to 0$.

We generated a movie under the assumption that spikes are generated from the marked homogeneous Poisson process with the constant rate. Fig 2 shows the estimation result. Although there are no relationship between spike trains and the covariate, Gaussian kernel used in our model approximates the constant rate function.

References

1. James LF. Bayesian calculus for gamma processes with applications to semiparametric intensity models. Sankhyā. 2003;65: 179–206.

2. Agulló J, Croux C, Aelst SV. The multivariate least-trimmed squares estimator. J Multivar Anal. 2008;99: 311–338.

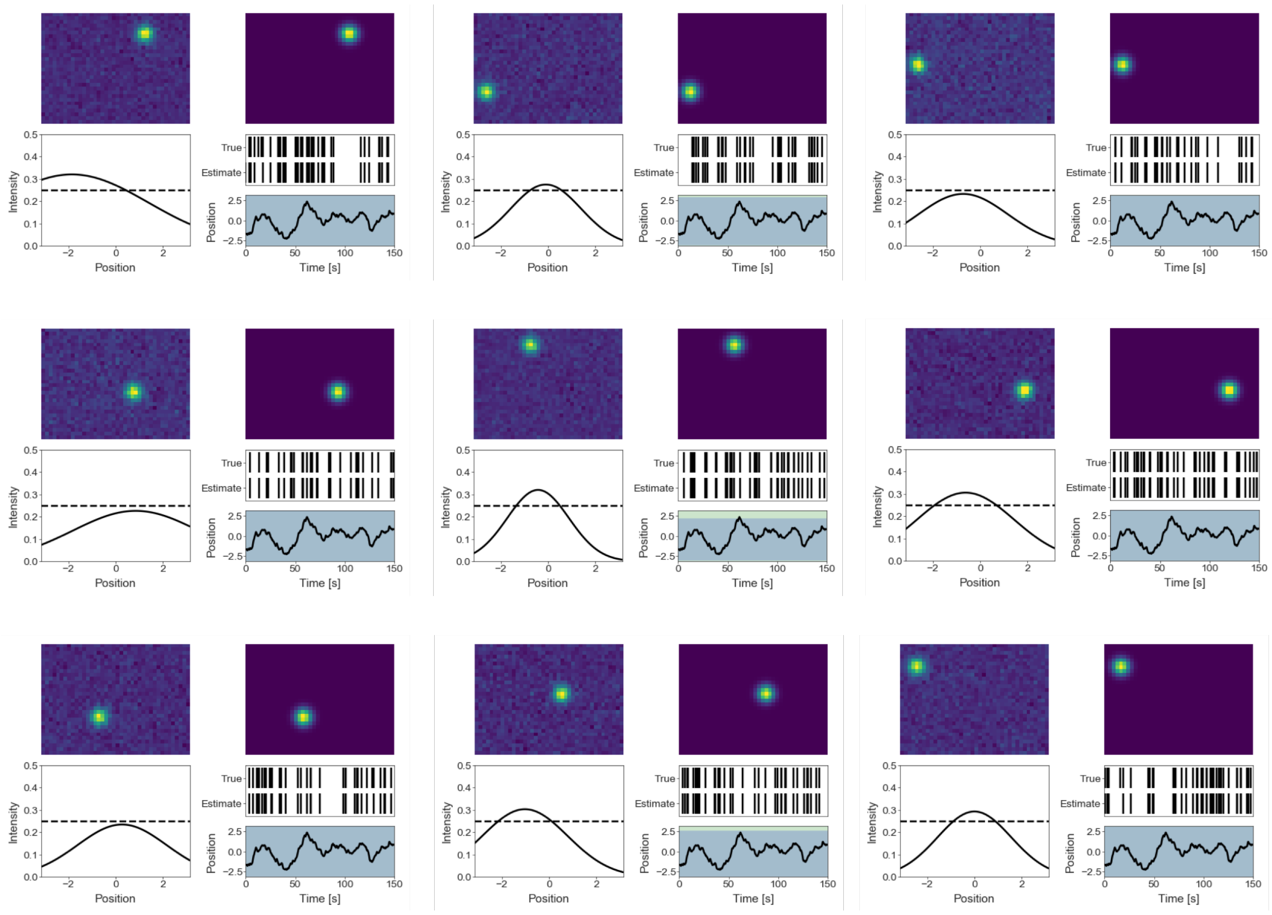

Fig 2: Estimation results obtained by our method in the simulation study. (Upper left) Estimated cell shape. (Upper right) True cell shape. (Bottom left) Tuning curve. The solid line shows the estimated tuning curve, and the dashed line shows the true tuning curve with the mean value closest to the estimated mean value μ_k^x. (Bottom right) Estimated spike train and calcium fluorescence. Vertical lines indicate when spikes occurred; the upper lines indicate true spikes, and the bottom lines indicate estimated spikes. The black line plot shows the external stimulus. The green band shows the receptive field decided by the true μ^x and τ^x, and the blue band shows the receptive field decided by the estimated values.
